# Comprehensive genomic analysis of *Bacillus subtilis* and *Bacillus paralicheniformis* associated with the pearl millet panicle reveals their antimicrobial potential against important plant pathogens

**DOI:** 10.1186/s12870-024-04881-4

**Published:** 2024-03-18

**Authors:** Mushineni Ashajyothi, Shivannegowda Mahadevakumar, Y. N. Venkatesh, Pullabhotla V. S. R. N. Sarma, Chalasani Danteswari, Alexander Balamurugan, Ganesan Prakash, Vikas Khandelwal, C. Tarasatyavathi, Appa Rao Podile, Kirankumar S. Mysore, Siddaiah Chandranayaka

**Affiliations:** 1 Plant Protection Lab, ICAR-Central Agroforestry Research Institute, Jhansi, Uttar Pradesh 284003 India; 2 Botanical Survey of India, Andaman and Nicobar Regional Centre, Haddo, Port Blair, Andaman and Nicobar Islands 744102 India; 3 Department of Plant Sciences, School of Life Sciences, University of Hyderabad, Hyderabad, Telangana 500046 India; 4 ICAR-Indian Agricultural Research Institute, New Delhi, 110012 India; 5 All India Coordinated Research Project On Pearl Millet, Agriculture University, Jodhpur, Rajasthan 342304 India; 6 Department of Biochemistry and Molecular Biology, Oklahoma State University, Stillwater, OK USA; 7 Department of Studies in Biotechnology, University of Mysore, Mysore, Karnataka 570 006 India

**Keywords:** Pearl millet, Genome, *Bacillus*, *Magnaporthe*, Seed-priming, Cyclic-lipo-peptide

## Abstract

**Background:**

Plant microbiome confers versatile functional roles to enhance survival fitness as well as productivity. In the present study two pearl millet panicle microbiome member species *Bacillus subtilis* PBs 12 and *Bacillus paralicheniformis* PBl 36 found to have beneficial traits including plant growth promotion and broad-spectrum antifungal activity towards taxonomically diverse plant pathogens. Understanding the genomes will assist in devising a bioformulation for crop protection while exploiting their beneficial functional roles.

**Results:**

Two potential firmicute species were isolated from pearl millet panicles. Morphological, biochemical, and molecular characterization revealed their identities as *Bacillus subtilis* PBs 12 and *Bacillus paralicheniformis* PBl 36. The seed priming assays revealed the ability of both species to enhance plant growth promotion and seedling vigour index. Invitro assays with PBs 12 and PBl 36 showed the antibiosis effect against taxonomically diverse plant pathogens (*Magnaporthe grisea; Sclerotium rolfsii; Fusarium solani; Alternaria alternata; Ganoderma* sp.) of crops and multipurpose tree species. The whole genome sequence analysis was performed to unveil the genetic potential of these bacteria for plant protection. The complete genomes of PBs 12 and PBl 36 consist of a single circular chromosome with a size of 4.02 and 4.33 Mb and 4,171 and 4,606 genes, with a G + C content of 43.68 and 45.83%, respectively. Comparative Average Nucleotide Identity (ANI) analysis revealed a close similarity of PBs 12 and PBl 36 with other beneficial strains of *B. subtilis* and *B. paralicheniformis* and found distant from *B. altitudinis, B. amyloliquefaciens,* and *B. thuringiensis*. Functional annotation revealed a majority of pathway classes of PBs 12 (30) and PBl 36 (29) involved in the biosynthesis of secondary metabolites, polyketides, and non-ribosomal peptides, followed by xenobiotic biodegradation and metabolism (21). Furthermore, 14 genomic regions of PBs 12 and 15 of PBl 36 associated with the synthesis of RiPP (Ribosomally synthesized and post-translationally modified peptides), terpenes, cyclic dipeptides (CDPs), type III polyketide synthases (T3PKSs), sactipeptides, lanthipeptides, siderophores, NRPS (Non-Ribosomal Peptide Synthetase), NRP-metallophone, etc. It was discovered that these areas contain between 25,458 and 33,000 secondary metabolite-coding MiBiG clusters which code for a wide range of products, such as antibiotics. The PCR-based screening for the presence of antimicrobial peptide (cyclic lipopeptide) genes in PBs 12 and 36 confirmed their broad-spectrum antifungal potential with the presence of *spoVG, bacA,* and *srfAA* AMP genes, which encode antimicrobial compounds such as subtilin, bacylisin, and surfactin.

**Conclusion:**

The combined in vitro studies and genome analysis highlighted the antifungal potential of pearl millet panicle-associated *Bacillus subtilis* PBs12 and *Bacillus paralicheniformis* PBl36. The genetic ability to synthesize several antimicrobial compounds indicated the industrial value of PBs 12 and PBl 36, which shed light on further studies to establish their action as a biostimulant for crop protection.

**Supplementary Information:**

The online version contains supplementary material available at 10.1186/s12870-024-04881-4.

## Introduction

Pearl millet [*Cenchurus americanus* (L.) R. Br.] is the sixth most predominant cereal crop cultivated both in arid and semi-arid tropical regions worldwide, grown for food and forage. India, being the largest producer of millets, accounts for 36% of the world’s millet production [1]. Pearl millet is grown on an area of 7.65 million hectares with an average production of 10.86 million metric tonnes during 2020–21 [2], and its standalone contribution was 40.51% of the total millet production from India. However, pearl millet is susceptible to several diseases [3–5], and blast disease induced by *Magnaporthe grisea* is a global threat with high genetic variability and increasing virulent races [6–8]. Since host plant resistance is vulnerable to new pathotypes and chemical pesticides are under the limelight, there is a need to develop an effective, alternative, environment-friendly sustainable disease management strategy.

Exploring the invisible, complex microbial community that includes a diverse and complex mixture of different species of microorganisms on and within plant spheres, which have subtle and relatively constant partner relationships can be a potential option for green technologies in plant disease management [9]. Plant microbiome, having evolutionary significance regulates the physiological functions of plant metabolism [10–15]. Plant microbiome members, unlike other microbiota, develop an intimate relationship with plant metabolism and influence it in harmony to alleviate various biotic and abiotic stress conditions. Among all, the firmicutes are well known for their abundance on plants, and the genus *Bacillus* has a special place with extensive studies on agroecology, including nutrient cycling [16–18], disease suppression [19–21], plant growth regulation [22, 23], diversity enhancement [24, 25] and phytoremediation [26, 27]. With this relevance, the pearl millet microbiome is under exploration of late, and associated microbes from the root, leaf, and stem regions of pearl millet are attributed in many studies to its drought stress tolerance ability [28]. Pearl millet leaf and root-originated *Bacillus subtilis*, *Bacillus pumilus* strains have shown their ability to enhance the shoot and root dry mass under phosphorous deficit conditions and are known to produce siderophores and Indole Acetic Acid, which benefit from withstanding adverse conditions [29]. Previously, pearl millet endophytic *Bacillus amyloliquefaciens* EPP90 was found to have multiple stress tolerance mechanisms and plant growth promotion activity [30]. However, little is known about the pearl millet microbiome's relation to foliar blast disease. Especially, the compact structure of the pearl millet spike and its exposure to extreme weather events challenge the spermosphere microbes to thrive on and withstand the stress. However, the spermosphere microbial functionality and influence on pearl millet growth and blat disease are less studied. In this study, two pearl millet panicle-associated *Bacillus* strains, PBs 12 and PBl 36, were identified and characterized for their plant growth promotion and broad-spectrum antifungal activity against pearl millet blast-causing *Magnaporthe grisea* as well as a few crop and tree pathogens. With the increasing public and government criticism on large-scale dependency of pesticides for plant disease management, this study will help in developing a suitable bioformulation for crop protection.

## Materials and methods

### Plant material and growth condition

The pearl millet genotype 7042S, which was raised in the experimental field at the University of Mysore, Manasagangotri (12.30° N, 76.64° E), Karnataka, India, was used in the present study. Panicle samples for isolation of spermosphere-associated microbes were collected at the physiological maturity stage (110–120 days) of the pearl millet in sterilized centrifuge tubes (50 ml), and the samples were immediately processed for panicle-associated microbe isolation under aseptic conditions in the laboratory at the Institution of Excellence, Vijnana Bhavana, University of Mysore, Manasagangotri.

### Isolation of pearl millet panicle-associated bacteria and morphotyping

Pearl millet panicle-associated epiphytic bacteria were isolated, according to Eyre et al. [31]. Briefly matured panicles from pearl millet plants (*n* = 12) were cut into two equal parts under aseptic conditions and transferred to the 500-ml glass bottles containing 300 ml of sterile 1X phosphate buffer saline with 0.1% Tween (PBST) at pH 7.2 and placed on an orbital shaker (180 rpm) for 30 min. The washed buffer alone (300 ml) was collected in new 500 ml bottles, and the washing step was repeated twice with 100 ml of PBST buffer. Pellets were harvested from panicle-washed buffer-microbe suspension and resuspended in 10 ml of PBS (PH-7.2) after centrifugation at 6000 rpm for 15–20 min. Serial dilutions made with sterile distilled water and 1 ml of 10^–4^, 10^–5^, 10–6, and 10^–7^ dilutions were poured onto nutrient agar (NA) media amended with 5% 2,3,5-triphenyl tetrazolium chloride (*n* = 3 replications for each dilution). Culture morphotyping was recorded 48 h after incubation at 28 °C. Well-separated, individual, distinct colonies were selected based on colony morphology and re-streaked onto fresh NA plates. Among several morphotypes on the NA surface, the two distinct colonies that displayed the most abundance were named PBs 12 and PBl 36, preserved at -80 °C for further characterization.

### Biochemical analysis of the pearl millet panicle-associated bacterial isolates PBs 12 and PBl 36

The pearl millet panicle-associated bacterial isolates PBs 12 and PBl 36 with distinct morphology were tested for their gram staining, gram-reaction (3% KOH test), siderophore production (CAS assay), citrate, potassium utilization, chitinase and lipase activity, starch utilization, ammonia production, and cellulase activity, respectively. Single colonies of 24-h-old culture were inoculated in the specific medium for all the biochemical tests. All the tests were conducted, and the observations were recorded as per the standard protocols [32, 33].

### DNA extraction and 16S rRNA sequencing

Genomic DNA was extracted from two representative isolates (PBs 12 and PBl 36) according to Chen and Kuo [34], and the qualitative and quantitative analysis of genomic DNA was performed using Nanodrop 2000 (Thermofisher, USA). Amplification of 16S rRNA was performed by Thermocycler (Eppendorf, Germany) using primers 27F (5’-AGAGTTTGATCCTGGCTCAG-3’) and 1492R (5’-GGTTACCTTGTTACGACTT-3’). PCR samples with genomic DNA were subjected to initial denaturation at 96 °C for 5 min, 35 cycles of denaturation at 95 °C for 60 s, annealing at 58 °C for 60 s, and extension at 72 °C for 60 s, followed by a final extension at 72 °C for 10 min. A 1% agarose gel electrophoresis with ethidium bromide was used to separate the PCR amplicons, and they could be seen on a QuantityOne UV transilluminator (BioRad, USA). Obtained amplicons of the 16S rRNA gene (~ 1500 bp) were used for bi-directional Sanger sequencing (Eurofins Scientific, India). The nucleotide sequences were assembled (> 1400 bp; DNA Baser assembler v5), trimmed using CLC sequence viewer 8.0.0 (https://clc-sequence-viewer.software.informer.com/8.0/), and curated sequences were compared with NCBI GenBank sequences (https://blast.ncbi.nlm.nih.gov/) based on the BLAST search tool (https://blast.ncbi.nlm.nih.gov/Blast.cgi). The curated sequences were finally submitted to NCBI GenBank via the submission portal BankIt (https://submit.ncbi.nlm.nih.gov/subs/genbank/) to generate accession numbers. The maximum likelihood method with the Tamura-Nei model [35] and 1000 bootstrap replications were used for the phylogenetic analysis. Similarity percentage was considered for species-level grouping of the isolates, wherein *Bacillus thuringiensis* species was considered an outgroup.

### Growth promotion activity of pearl millet panicle associated *Bacillus* strains PBs 12 and PBl 36

The effect of pearl millet panicle associated with two *Bacillus* strains, PBs 12 and PBl 36, on seed germination, enhancing seedling growth parameters including shoot and root length, and seedling vigour index, on pearl millet cv. 7042S, was investigated by a seed priming assay [36]. *Bacillus* strains PBs 12 and PBl 36 were grown on NA media, and a 24-h-old culture was used for the preparation of a 1.0 OD (1 × 10^8^ cfu/mL) bacterial suspension. Pearl millet cv. 7042S seeds were soaked in bacterial suspension overnight and later allowed to grow for 7 days under climate-controlled greenhouse conditions at 28 °C with 90% relative humidity. Similar treatments with sterile water alone served as controls, and four replications were maintained for each treatment separately. The growth parameters, including germination percentage, root, and shoot length, were recorded, whereas seedling vigour was calculated using the given formula [37].

$$\mathrm{Seed}\;\mathrm{vigour}\;\mathrm{index}=\mathrm{Germination}\;\mathrm{percentage}\;(\%)\;\times\;\mathrm{Seedling}\;\mathrm{length}\;(\text{mm})$$

Data analysis was performed using the raw data from all the recorded parameters separately for statistical ANOVA analysis, applying the Completely Randomized Design (CRD) through the WASP 2.0 (Web Agri Stat Package) software, which can be accessed at http://www.ccari.res.in/wasp2.0/index.php. The mean values have been grouped based on Critical Difference (CD) to indicate the significance at probability (*p*) = 0.05%

### Dual-culture confrontation assay of *Bacillus* strains PBs 12 and PBl 36 with taxonomically diverse fungal plant pathogens

Dual-culture confrontation assays were carried out on potato dextrose agar medium as described by Dennis and Webster [38]. The following 6 fungal plant pathogens, comprising *Magnaporthe grisea* (foliar blast of pearl millet), *Sclerotium rolfsii* (stem rot of tomato), *Fusarium solani* (root rot and wilt in malabar neem), *Ganoderma* spp. 1 and 2 (wood rots in *Tectona grandis* and *Pongamia pinnata),* and *Alternaria alternata* (leaf blight of *Tectona grandis)* were used for in vitro screening with *Bacillus* strains PBs 12 and PBl 36, respectively. Bacterial cultures were grown on NA media, and a 24-h-old culture was used for the dual-culture assay. Mycelial discs of 0.5 mm from 7-day-old fungal cultures placed on PDA plates and loops of bacterial culture streaked on the opposite side of the same Petri plate were perpendicular to each bacterial isolate, whereas the fungal disc alone in the media served as the control. Three replications were maintained for all the treatments with each fungus, and inoculated plates were incubated at 26 ± 1 °C for 7 days. When the control plates attained full mycelial growth, the radial growth of the fungal pathogens was calculated, and percent inhibition over control was estimated by using the given formula [39].

$$\mathrm{Percent}\;\mathrm{mycelial}\;\mathrm{inhibition}\;\mathrm{over}\;\mathrm{control}=(\mathrm{Mycelial}\;\mathrm{growth}\;\mathrm{in}\;\mathrm{mockplate}-\mathrm{Mycelial}\;\mathrm{growth}\;\mathrm{in}\;\mathrm{treatedplate})/\;\mathrm{Mycelial}\;\mathrm{growth}\;\mathrm{in}\;\mathrm{mockplate}\rbrack\ast100\rbrack$$

The inhibition percentage values were further analyzed by statistical ANOVA, applying the Completely Randomized Design (CRD) through the WASP 2.0 (Web Agri Stat Package). The mean values have been grouped based on Critical Difference (CD) to indicate the significance at probability (*p*) = 0.05%. The experiment was repeated twice to confirm the reproducibility of the results.

## Whole genome sequencing of *Bacillus* strains PBs 12 and PBl 36

### Whole genome sequencing of *Bacillus subtilis* PBs12 and *Bacillus paralicheniformis* PBl 36

#### Strain and DNA extraction

The bacterial strains *Bacillus subtilis* PBs 12 and *Bacillus paralicheniformis* PBl36 were isolated, DNA extraction was performed as described above, and the identity was re-confirmed by sequencing 16S rDNA [27F- 5’-AGAGTTGATCCTGGCTCAG- 3’ and 1492R- 5’-GGTTACCTTGTTACGACTT-3’]. Briefly, the bacterial culture (100 -µg mL^−1^) was grown on Nutrient agar media [NA, gL^−1^ Peptone 5.0; Beef Extract 3.0; NaCl 5.0; Agar 15.0; pH 7.0 ± 0.2] and incubated at 28 °C for 48 h. The fresh culture was washed with 0.5% NaCl used for DNA extraction using the CTAB method. DNA quality check was performed using Nanodrop and agarose gel electrophoresis (0.8% Agarose Gel) respectively.

#### Library preparation and QC

The samples that passed quality control were selected for further processing in the library preparation step. To summarize, we used 100 nanograms of DNA to create libraries with unique identifiers using the Truseq Nano library preparation kit from Illumina (#20015964). The final libraries were assessed for concentration using a Qubit 4.0 fluorometer from Thermofisher (#Q33238) and a DNA HS assay kit (#Q32851), following the provided instructions. We determined the size and base length of the fragmented DNA libraries by running them on an Agilent Tapestation 4150 system with highly sensitive D1000 screen tapes (#5067–5582) according to the manufacturer's protocol.

Following the sequencing, we initially examined the data for base quality and the presence of adapters using the Fast QC (https://www.bioinformatics.babraham.ac.uk/projects/fastqc/) approach. To remove adapters and trim the sequence ends, a trimmomatic (http://www.usadellab.org/cms/uploads/supplementary/Trimmomatic/TrimmomaticManual_V0.32.pdf) analysis was performed.

#### Genome assembly, molecular phylogenetic analysis and annotation

The paired-end files were imported for analysis, and a De novo genome assembly was performed using CLC Genomics Workbench version 12.0. The resulting assembled genome served as the basis for additional scaffolding performed on the Medusa server (http://combo.dbe.unifi.it/medusa/). The final scaffold files of both PBs 12 and PBl 36 draft genomes were utilized in subsequent steps, including molecular phylogeny and functional annotation analysis. To establish comparative genomic relationships, the OrthoANI algorithm was employed to calculate average nucleotide identity (ANI), and a UPGMA tree was generated by aligning whole genomes. This analysis included reference sequences for *Bacillus subtilis* (NCIB3610, NBRC 13719, DSM 10, KCTC 3135, 168); *Bacillus paralicheniformis* (A4-3, PRO 109, MDJK 30, Bac 84, ATCC 14580) and other *Bacillus* species *(B. halotolerans, B. velezensis, B. amyloliquefaciensis, B. pumilus, B. thuringiensis)* as out groups [40].

The assembled genome was subsequently annotated using the Pathosystems Resource Integration Centre (PATRIC) platform (https://www.patricbrc.org/). PATRIC facilitated the extraction of information related to sequence length, N50, G + C content, a circular view of the genome, pathway summaries, and cellular subsystems. Additionally, a comprehensive analysis was conducted to identify biosynthetic gene clusters responsible for producing secondary metabolites in the *Bacillus* strains PBs 12 and PBl 36. This analysis was carried out using the antiSMASH server (Antibiotics and Secondary Metabolite Analysis Shell) version 4.1.0 (http://antismash.secondarymetabolites.org) as detailed by Medema et al. [41] and Blin et al. [42]. Finally, the whole genome data of *Bacillus subtilis* PBs 12 and *Bacillus paralicheniformis* PBl 36 was submitted to the NCBI GenBank through the genome submission process under Biosample IDs: SAMN31359864 and SAMN31577901.

## Results

### Morphological and biochemical characterization of two pearl millet panicle microbiome associated *Bacillus* strains PBs 12 and PBl 36

The pearl millet panicle-associated microbiota was dominated by the genera *Bacillus,* comprising *Bacillus licheniformis, Bacillus paralicheniformis*, *Bacillus subtilis*, *Bacillus megaterium,* and *Bacillus aryabhattai,* apart from other important bacterial species like *Pantoea stewartii* and *Sphingomonas sanguinis*. Among all, two isolates were observed as the most abundant *Bacillus* species that displayed the highest antifungal activity**.** The two representative isolates were named PBs 12 and PBl 36 and considered for further characterization.

The colony morphology of *Bacillus* sp. PBs 12 was observed as round to irregular pale creamy white colonies with a shiny surface and diffused irregular margins, while *Bacillus* sp. PBl 36 produced irregular pale white/ash-colored colonies with spreading margins and rough surface (Table 1). The biochemical analysis revealed that the bacterial isolates are gram-positive, rod-shaped and did not produce fluorescence on King’s B media. They also lack chitinase and lipase activity and can grow at 37°C within 24 h. These bacterial isolates varied in their citrate utilization ability, starch hydrolysis, siderophore production, and ammonia production. *Bacillus subtilis* PBs 12 tested positive for siderophore production, able to utilize citrate but lacking lipase and chitinase activity. Both PBs 12 and PBl 36 were found to have cellulase activity and were negative for indole production and nitrate utilization tests (Supplementary Table 1).

**Table 1:** The identity of pearl millet panicle microbiome member species *Bacillus subtilis* and *Bacillus paralicheniformis* based on morphology and 16S r RNA gene sequencing

**Bacterial Species**	**Isolate**	**GenBank Accession no**	**16S r RNA Length (bp)**	**Colony characters**	**Identity (%)**	**Closest match in NCBI data base**	**Colony morphology of *C. americanus* panicle associated bacteria**
							**TZC non-amended nutrient agar media**
*Bacillus subtilis*	PBs 12	OL674150	1416	Round to Irregular pale creamy white colonies with shiny surface and diffused irregular margins	98.31	MT641205.1	
*Bacillus paralicheniformis*	PBl 36	OL674149	1416	Irregular pale white colonies with spreading margin and rough surface	99.44	MT184872.1	

**Fig. 1:**
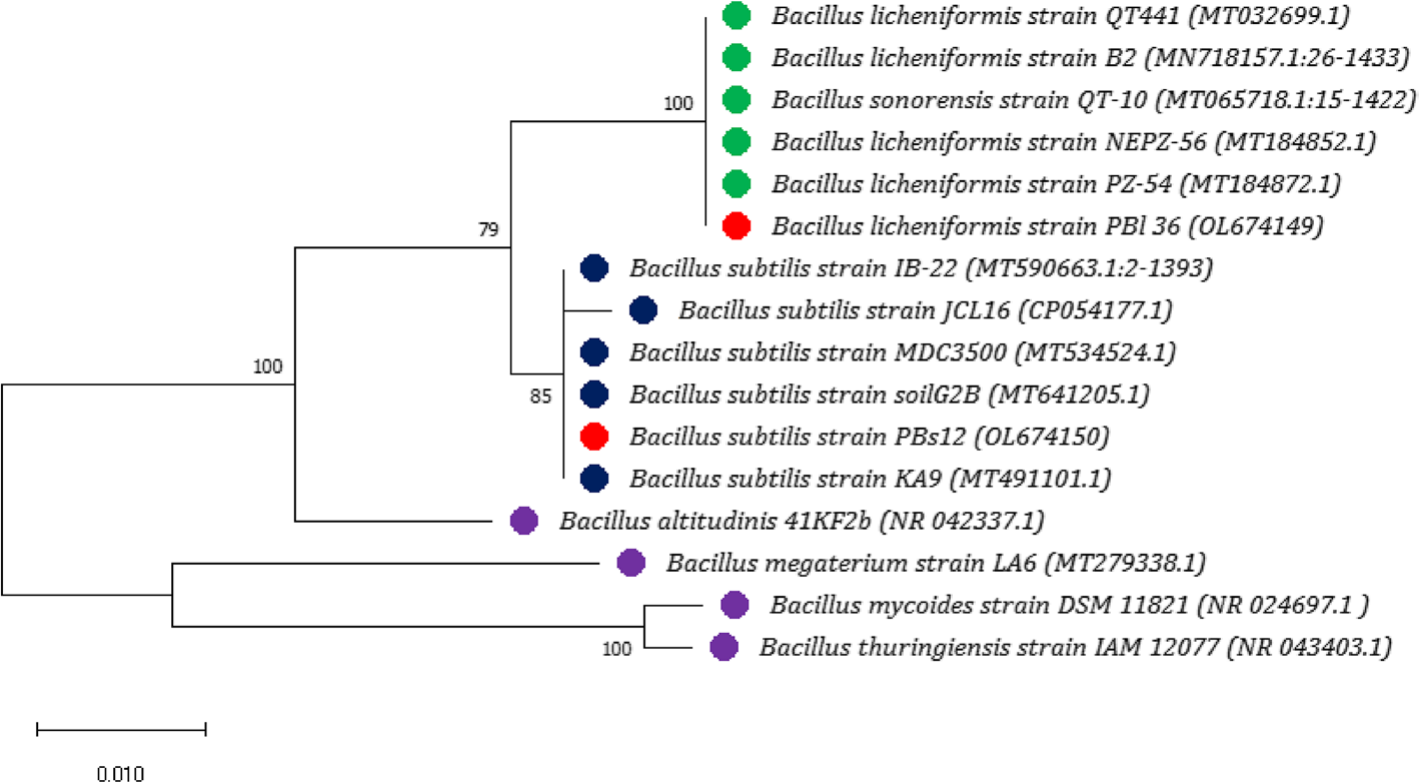
16S ribosomal RNA based phylogenetic analysis of pearl millet panicle associated *Bacillus subtilis* PBs 12 and *Bacillus licheniformis* PBl 36 Maximum-likelihood tree constructed based on Tamura-Nei model (1993) for *Bacillus* strains ( *Bacillus subtilis* PBs 12 and *Bacillus licheniformis* PBl 36) derived from MUSCLE alignment of 16S ribosomal RNA partial sequences [Boot strap values are based on 1000 repetitions and *Bacillus thuringiensis* strain IAM12077 considered as an outgroup]

The 16S ribosomal RNA sequence (1416 bp) of representative *Bacillus* isolates, PBs 12 and PBl 36 were checked for low-quality bases, and the contigs were end-trimmed and curated using a CLC sequence viewer. The BLAST analysis in the NCBI Nucleotide BLAST database using the 16S r RNA-curated sequence of PBl 36 revealed its close similarity with the wheat seed endophyte, *Bacillus licheniformis* strain PZ-54 from New Delhi, India (MT184872.1), with a 99.44% identity. Similarly, PBs 12 revealed its close similarity with *Bacillus subtilis* strain soil G2B (MT184872.1) from Gujarat, India, with 98.31% identity. Curated sequences of 16S r RNA from both PBs 12 and PBl36 were submitted to the NCBI GenBank (Table 1). The accession numbers allotted were as follows for *Bacillus subtilis* PBs 12 (accession no.: OL674150) and *Bacillus licheniformis* PBl 36 (accession no.: OL674149). Phylogenetic analysis using 16S ribosomal RNA sequence based on the maximum likelihood tree construction method revealed that isolate PBs 12 clustered with *Bacillus subtilis* strains and PBl 36 closely clustered with the other *Bacillus licheniformis strains.* Both *Bacillus licheniformis* PBl 36 and *Bacillus subtilis* PBs 12 were distant from other *Bacillus* species such as *Bacillus megaterium*, *Bacillus altitudinis*, *Bacillus mycoides* and *Bacillus thuringiensis* (Fig. 1).

### Effect of pearl millet panicle-associated *Bacillus* strains PBs 12 and PBl 36 on pearl millet cv. 7042S seedling growth

*Bacillus* strains PBs 12 and PBl 36 were used for seed priming to test their efficacy on plant growth promotion. Germination percentage (%), seedling vigour index, seedling shoot, and root length were estimated to find the growth promotion potential. Seed priming with PBs 12 enhanced the seedling vigour index up to 1186.55 with the enhanced seedling shoot (6.87 cm) and root length (5.62 cm), while PBl 36 showed maximum plant growth promotion activity with the highest seedling vigour index (1324.8), shoot length (7.38 cm), and root length (6.42 cm). In sterile water-treated mock plants, seedling shoots and root length were 5.01 and 5.56 cm, respectively, and seedling vigour index was recorded as 1004.15 (Table 2), indicating both the *Bacillus* isolates have a considerable plant-growth-promoting function.

**Table 2:** Seed priming with pearl millet panicle associated *Bacillus* strains PBs 12 and PBl 36 to test their plant growth promotion activity

**Isolate name**	**Shoot length**	**Root length**	**Germination percentage (%)**	Seedling vigour index*
***Bacillus subtilis* PBs 12**	6.87b	5.62b	95	1186.55
***Bacillus licheniformis* PBl 36**	7.38a	6.42a	96	1324.80
**Mock**	5.01c	5.56b	95	1004.15
**CD (*p* = 0.05%)**	0.49	0.22	-	-

Data sharing the same letter designation in each column, indicate no significant difference between them at a significance level of *p* = 0.05

CD (Critical Difference): Smallest difference between treatments means likely to indicate true change

24 h old bacterial cultures of PBs12 and PBl 36 used to prepare 1 OD (1 × 10^8^ cfu/ml) cell suspension. Pearl millet cv. 7042S seeds (*n* = 100) were soaked overnight in cell suspensions and incubated for 7 days at 27^o^ C temperature and 98% relative humidity. Data recoded and interpreted 7 days after incubation

Seedling vigour index was calculated using the formula:

$$\mathrm{Seedling vigour index}* = [(\mathrm{Seedling length}) * (\mathrm{Germination percentage})]$$

### Antifungal activity of pearl millet panicle associated *Bacillus* strains PBs 12 and PBl 36

**Fig. 2:**
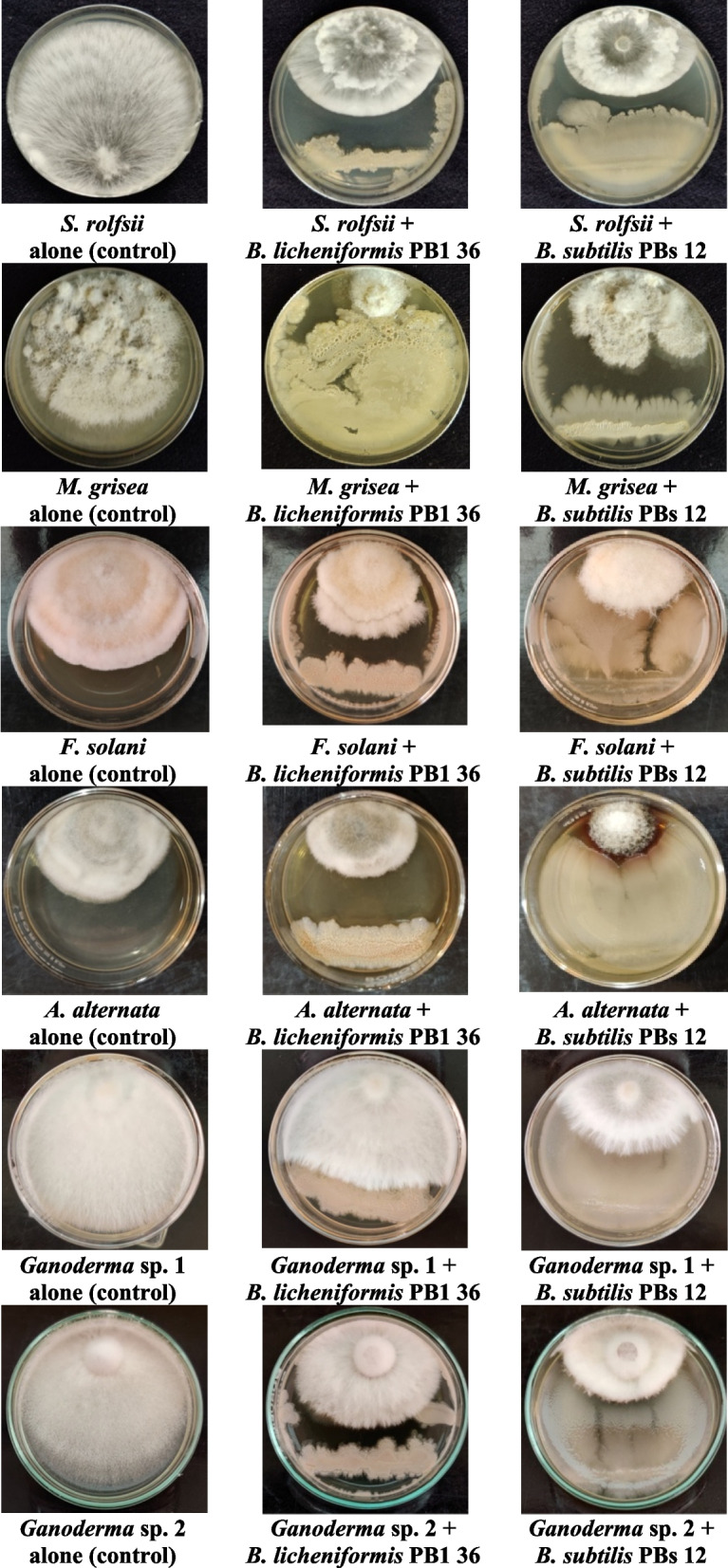
In-vitro screening of pearl millet panicle associated Bacillus isolates PBs 12 and PBl 36 against diverse fungal plant pathogens. Note: Dual culture assays to estimate the bio-control activity of bacterial strains associated with panicles of Cenchrus americanus, performed on sterile Potato Dextrose Agar plates with six taxonomically diverse fungal plant pathogens. 48 h old pure bacterial culture [column 3 (**b**); 3 (**c**)] used for bacterial suspension (1 × 10^7^ cfu/ml) preparation. Loopful of culture steaked one side of the Petri plate (90 mm) and mycelial disc (4 mm dia.) placed opposite side confronting the bacteria. 3 replications maintained for each isolate and culture plates without bacteria and inoculated with only fungal pathogens served as mock [column: 3 (**a**))]. All the plates incubated at 28° C until the mycelium cover mock plate completely, then the inhibition zone measured and average of 3 replications considered as final radial mycelial growth reading. Experiment repeated twice to check the reproducibility

Several *Bacillus* species are well known for their antimicrobial activity. The strains PBs 12 and PBl 36 were tested against taxonomically diverse plant pathogens and found effective in the suppression of their mycelial growth. Dual-culture confrontation assays against diverse fungal plant pathogens comprising *Magnaporthe grisea* (foliar blast of pearl millet), *Sclerotium rolfsii* (stem rot of tomato), *Fusarium solani* (root rot and wilt in malabar neem), *Ganoderma* spp. 1 and 2 (wood rots in *Tectona grandis* and *Pongamia pinnata*) and *Alternaria alternata* (leaf blight of *Tectona grandis*) revealed that the tested *Bacillus* strains were able to effectively inhibit the mycelial growth up to 25 to 78% (Table 3). *Bacillus subtilis* PBs 12 (60.4 – 78.70%) showed strong antifungal activity and inhibited > 60% of mycelial growth in all test pathogens, with a minimum inhibition percentage of 60.40 against *Magnaporthe grisea* and a maximum of 78.70% against *Alternaria alternata* followed by *Bacillus licheniformis* PBl 36, which recorded the lowest inhibition percentage (25%) against *Ganoderma* spp. and the maximum (69.5%) against *Athelia rolfsii.* However, both the isolates have proven to have strong antifungal activity against taxonomically diverse plant pathogens from both crops and trees (Fig. 2).

**Table 3:** Ability of pearl millet panicle associated *Bacillus licheniformis* PBl 36 and *Bacillus subtilis* PBs 12 on mycelial growth inhibition of taxonomically diverse plant pathogens in Invitro dual culture assays

**Disease**	**Fungal species**	**Phylum**	**Order**	**Family**	**Mycelial growth inhibition (%)**		
					***Bacillus licheniformis* PBl 36**	***Bacillus subtilis* PBs 12**	**Mock**
**Southern corn leaf blight**	*Athelia rolfsii*	Basidiomycota	Atheliales	Atheliaceae	69.54a	71.16b	0.00f
**Pearl millet Blast**	*Magnaporthe grisea*	Ascomycota	Magnaporthales	Magnaporthaceae	54.64b	60.40c	0.00f
**Malabar neem seedling wilt**	*Fusarium solani*	Ascomycota	Hypocreales	Nectriaceae	56.00b	70.00b	0.00f
**Teak leaf blight**	*Alternaria* sp.	Ascomycota	Pleosporales	Pleosporaceae	43.40d	78.70a	0.00f
**Teak basal rot**	*Ganoderma* spp. 1	Basidiomycota	Polyporales	Ganodermataceae	25.36e	61.73c	0.00f
**Pongamia wood rot**	*Ganoderma* spp. 2	Basidiomycota	Polyporales	Ganodermataceae	52.40c	75.77a	0.00f
**CD (*p* = 0.05%)**	*-*	-	-	-	2.24	3.13	-

Data sharing the same letter designation in each column, indicate no significant difference between them at a significance level of *p* = 0.05

CD (Critical Difference): Smallest difference between treatments means likely to indicate true change

Mycelial growth was recorded once the mock plate attained full petri plate coverage of each inoculated fungal species here *Athelia rolfsii* (3 days); *Magnaporthe grisea* (10 days); *Fusarium oxysporum* (6 days); *Alternaria* sp. (6 days); *Ganoderma* spp. 1 (3 days); *Ganoderma* spp. 2 (4 days). Mycelial growth inhibition was calculated using the formula: [(Mycelial growth in mock plate—Mycelial growth in treated plate)/ Mycelial growth in mock plate]*100

### Whole genome sequencing, assembly, and annotation

**Fig. 3:**
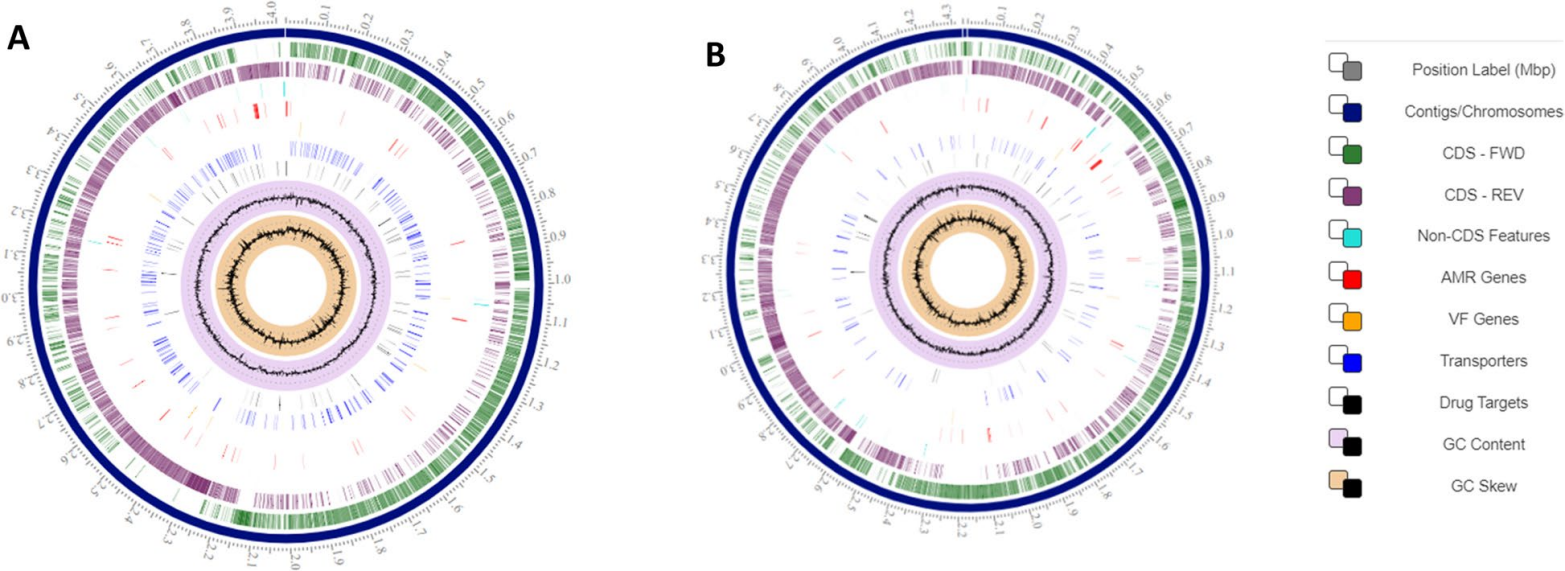
Circular genome view of pearl millet associated *Bacillus sp. ****a**** Bacillis subtilis* PBs 12; b) *Bacillus licheniformis* PBl 36

The first complete genome sequence of pearl millet panicle microbiome member species *Bacillus subtilis* PBs 12 and *Bacillus paralicheniformis* PBl 36 with growth promotion and antimicrobial potential against diverse plant pathogens has been unveiled. *Bacillus subtilis* PBs 12 (CP110213) has a 4020447 bp circular chromosome, which consists of 4171 genes with 43.68% G + C content, while *Bacillus paralicheniformis* PBl 36 (JAPEZR000000000) consists of a 4339481 bp circular chromosome, which consists of 4606 genes with 45.83% G + C content (Table 4). A circular genome view (Fig. 3) of both PBs12 and PBl 36 is presented, where contigs/chromosomes, CDS, non-CDS, AMR (antimicrobial resistance) genes, VF (virulence factor) genes, transporters, drug targets, GC content, and GC skew are depicted from the outer to the inner ring with different colour codes.

**Table 4:** Genome statistics of pearl millet associated *Bacillus subtilis* and *Bacillus paralicheniformis*

**Genome Parameters**	***Bacillus subtilis* PBs12**	***Bacillus paralicheniformis* PBl 36**
Sequencing Platform	Illumina Hiseq-2500	Illumina Hiseq-2500
Genome size	4020447 (bp)	4339481 (bp)
G + C content (%)	43.68	45.83
Number of scaffolds	01	02
Scaffold N50	4020447	4328796
No of genes/cds	4171	4606
PLFAM CDS	3961	4363
rRNA	05	06
tRNA	76	77
Pathways	136	135
Subsystems	289	289

### Molecular phylogenetic analysis of *Bacillus* strains PBs12 and PBl36 using whole genome sequences, with a focus on average nucleotide identity

**Fig. 4:**
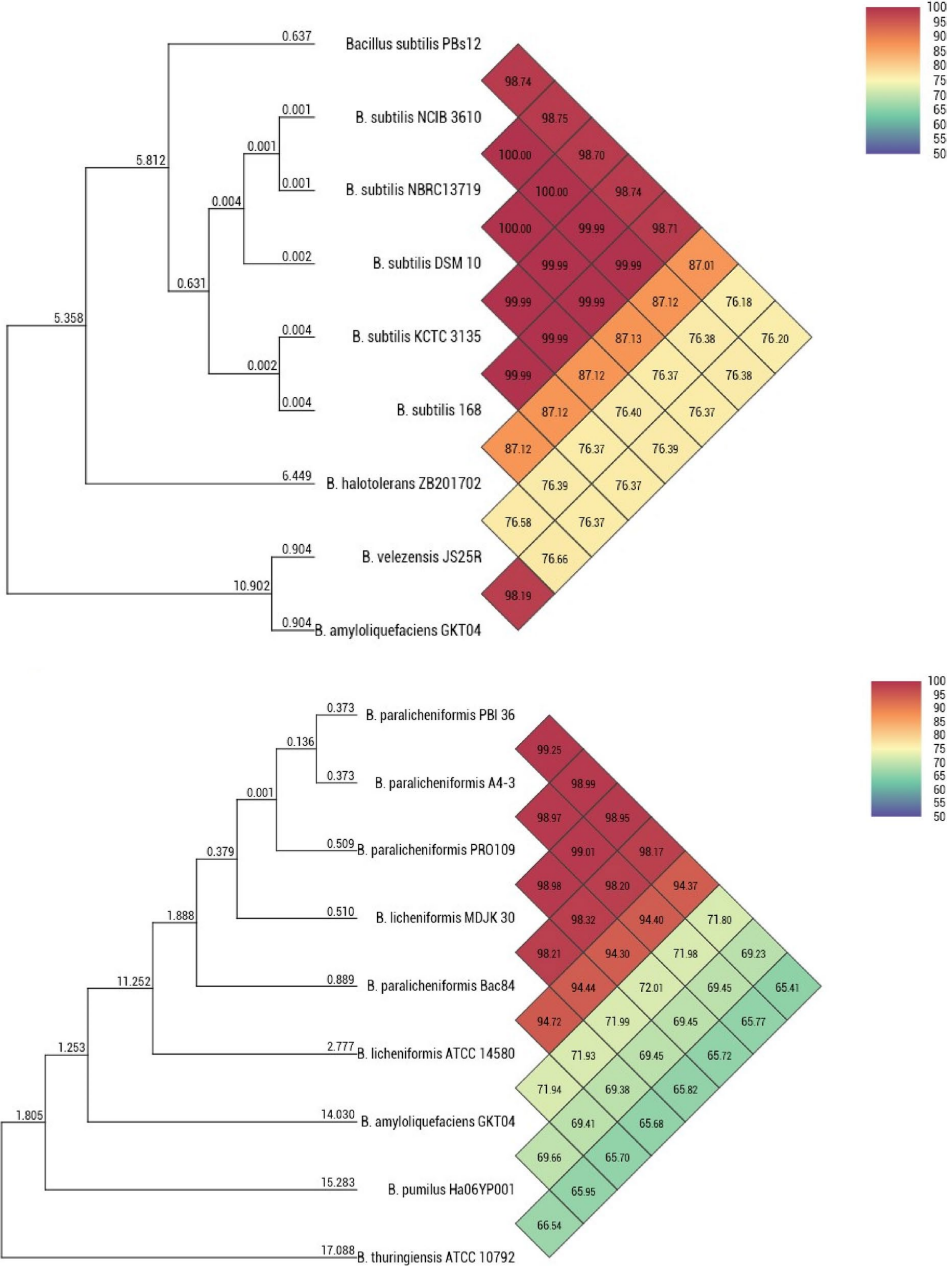
Average Nucleotide Identity among genomes of *Bacillus* species. Note: Comparative genome analysis revealed *Bacillus subtilis* PBs12 and ***Bacillus**** paralicheniformis* PBl36 have > 99% average nucleotide identity (ANI) with respective *Bacillus* species and distant from *Bacillus thuringiensis, Bacillus pumilus, Bacillus amyloliquefaciens, Bacillus halotolerans, Bacillus velezensis* with 65 to 76% ANI

The comparative genome average nucleotide identity (ANI) was calculated using the OrthoANI algorithm for phylogenetic analysis. The average nucleotide identity analysis was performed between whole genome sequences of *Bacillus subtilis (*PBs 12, NCIB3610, NBRC 13719, DSM 10, KCTC 3135, 168)*; Bacillus paralicheniformis (*PBl 36, A4-3, PRO 109, MDJK 30, Bac 84, ATCC 14580); and other *Bacillus species (B. halotolerans, B. velezensis, B. amyloliquefaciens, B. pumilus, B. thuringiensis*) as out groups (Table 5). The strain *Bacillus subtilis* PBs 12 closely clustered with *Bacillus subtilis* strains (ATCC12633, NCIB3610, NBRC 13719, DSM 10, KCTC 3135, 168) and *Bacillus paralicheniformis* PBl *36* with A4-3 and PRO 109 and showed 98.70% and 99% average nucleotide identity, respectively. Among all, the *Bacillus subtilis* PBs12 showed close association with the strain NBRC 13719 of Japan, with the highest (98.75%) identity. Similarly, PBs 12 shared 76.18 to 87.01% identity with *B. halotolerans, B. velezensis, and B. amyloliquefaciens,* which were distant from *Bacillus subtilis* strains in the phylogenetic tree (Fig. 4). On the other hand, *Bacillus paralicheniformis* PBs 12 was very similar to the strain A4-3 from South Korea, sharing 99.25% identity. It was also similar to *B. amyloliquefaciens*, *B. pumilus*, and *B. thuringiensis*, but not to other *Bacillus paralicheniformis* strains. The comparative ANI analysis for each genome combination has been given to show a distinct relationship between strains (Supplementary Table 2).

**Table 5:** Genome features of *Bacillus subtilis* and *Bacillus paralicheniformis*

Strain	Bio Project	Country	Isolation source	Genome size	GC %	CDS
*Bacillus subtilis*						
PBs12	PRJNA891890	India	*Pennisetum glaucum*	4.02	43.68	4171
NCIB 3610	PRJNA377766	USA	NA	4.30	43.00	4333
NBRC 13719	PRJDB8042	Japan	NA	4.30	43.00	4357
DSM 10	PRJNA941188	Denmark	NA	4.30	43.00	4315
KCTC 3135	PRJNA302835	Korea	NA	4.20	43.50	4258
B. subtilis 168	PRJNA846324	China	CCTCC	4.20	43.50	4253
*Bacillus paralicheniformis*						
PBl36	PRJNA897098	India	*Pennisetum glaucum*	4.33	45.83	4363
A4-3	PRJNA552192	South Korea	*Tomato*	4.60	45.50	4438
PRO 109	PRJNA941188	Ghana	Kantong	4.60	45.50	4488
MDJK 30	PRJNA379336	China	Peony rhizosphere	4.40	45.50	4134
Bac84	PRJNA412146	Saudi Arabia	Red Sea lagoons-Microbial mat	4.40	45.50	4285
ATCC 14580	PRJNA509976	China	NA	4.20	46.00	4199

Source: NCBI (National Center for Biotechnology Information) https://www.ncbi.nlm.nih.gov

### Functional genome annotation of *Bacillus subtilis *PBs 12 and *Bacillus paralicheniformis* PBl 36

Functional annotation of the assembled genome was performed using PATRIC, the bioinformatics resource centre (BRC) approach (https://www.bv-brc.org/). A total of 136 pathway classes in *Bacillus subtilis* PBs 12 and 134 in *Bacillus paralicheniformis* PBl 36 available in the genome. Approximately 50% of the pathway classes are involved in the biosynthesis of secondary metabolites (30 and 29 in each) followed by xenobiotics biodegradation and metabolism (21), carbohydrate metabolism (15), lipid metabolism (14), amino acid metabolism (13), metabolism of cofactors and vitamins (12). Two pathways were found related to signal transduction, one with the immune system and others related to various metabolic activities (Table 6). The pathway summary for all the pathway classes is presented along with the information on the number of genes annotated in Supplementary Table 3. To further investigate the biocontrol potential of PBs 12 and PBl 36, the pathways classes related to secondary metabolite biosynthesis, antifungal/antimicrobial compounds secretion, plant disease resistance priming and biodegradation of heavy metals were analyzed. The focus on selected pathways such as biosynthesis of polyketides and non-ribosomal peptides, biosynthesis of secondary metabolites, signal transduction and xenobiotics biodegradation revealed the ability of the PBs 12 and PBl 36 genome for several antimicrobial compounds (Table 7). Under the pathway class, biosynthesis of polyketides and non-ribosomal peptides found in both PBs 12 and PBl 36, while polyketide sugar unit biosynthesis was observed only in PBs 12. In the second class, biosynthesis of secondary metabolite pathways involved in caffeine metabolism, several antibiotics, isoquinoline alkaloid biosynthesis, terpenoid backbone biosynthesis, phenylpropanoid biosynthesis etc. The phosphatidylinositol signaling system and mTOR signaling pathway were found under the signal transduction class. Similarly, both PBs 12 (atrazine, naphthalene, tetrachloroethane, anthracene, benzoate, caprolactam, hexachlorocyclohexane, geraniol, styrene) and PBl 36 (1,4-Dichlorobenzene, benzoate, biphenyl, hexachlorocyclohexane, geraniol, styrene, toluene) showed xenobiotics biodegradation related metabolism indicating their bioremediation potential (Table 7). Finally, the PBs 12 and PBl 36 showed 289 subsystems distributed in 11 subsystem superclasses (Supplementary Table 4) with a total of 1,799 and 1,850 genes in each genome. Among which 35 subsystems are directly involved in stress response, defense and virulence with 136 and 124 genes.

**Table 6:** Summary of pathway classes in pearl millet adapted *Bacillus subtilis* and *Bacillus paralicheniformis* genomes

**Pathway_class**	***Bacillus subtilis* PBs12**	***Bacillus paralicheniformis* PBl 36**
Amino Acid Metabolism	13	13
Biosynthesis of Polyketides and Nonribosomal Peptides	07	06
Biosynthesis of Secondary Metabolites	23	23
Carbohydrate Metabolism	15	15
Energy Metabolism	07	07
Glycan Biosynthesis and Metabolism	09	09
Immune System	01	01
Lipid Metabolism	14	14
Metabolism of Cofactors and Vitamins	12	12
Metabolism of Other Amino Acids	09	09
Nucleotide Metabolism	02	02
Signal Transduction	02	02
Translation	01	01
Xenobiotics Biodegradation and Metabolism	21	20
**Grand Total**	**136**	**134**

**Table 7:** Pathways involved in secondary metabolite biosynthesis and bioremediation in *Bacillus* strains PBS12 and PBl36

**Pathway Class**	**Pathway Name_*B. subtilis* PBS12**	**EC Number**	**Pathway Name_*B. paralicheniformis* PBl36**	**EC Number**
Biosynthesis of Polyketides and Nonribosomal Peptides	Polyketide sugar unit biosynthesis	2.7.7.24; 4.2.1.46	Biosynthesis of siderophore group nonribosomal peptides	3.3.2.1; 2.7.7.58; 1.3.1.28
	Biosynthesis of siderophore group nonribosomal peptides	1.3.1.28; 2.7.7.58; 3.3.2.1	-	-
	Biosynthesis of ansamycins	2.2.1.1	-	-
Biosynthesis of Secondary Metabolites	Streptomycin biosynthesis	1.1.1.133; 5.1.3.13; 2.7.1.2; 3.1.3.25	beta-Lactam resistance	3.5.2.6
	Caffeine metabolism	1.7.3.3; 1.14.14.1	Caffeine metabolism	1.14.14.1
	Betalain biosynthesis	1.10.3.-	Isoquinoline alkaloid biosynthesis	2.6.1.1
	Tetracycline biosynthesis	6.4.1.2	Limonene and pinene degradation	1.14.13.-
	Terpenoid backbone biosynthesis	2.5.1.30; 2.7.1.148; 1.1.1.267; 2.5.1.31; 2.5.1.10; 5.3.3.2; 4.6.1.12; 2.7.7.60	Novobiocin biosynthesis	1.3.1.12
	Isoquinoline alkaloid biosynthesis	2.6.1.57	Penicillin and cephalosporin biosynthesis	3.5.1.11; 3.1.1.41
	Carotenoid biosynthesis	2.5.1.96	Phenylpropanoid biosynthesis	1.1.1.195
	Penicillin and cephalosporin biosynthesis	3.1.1.41	Terpenoid backbone biosynthesis	4.6.1.12; 1.1.1.267; 2.5.1.30; 2.5.1.10; 2.5.1.31; 5.3.3.2; 2.7.7.60; 2.7.1.148
	Penicillin and cephalosporin biosynthesis	3.5.2.6	Zeatin biosynthesis	2.5.1.75
	Zeatin biosynthesis	2.5.1.75	-	-
	Phenylpropanoid biosynthesis	1.1.1.195	-	-
Signal Transduction	Phosphatidylinositol signaling system	2.7.7.41	Phosphatidylinositol signaling system	2.7.7.41
	mTOR signaling pathway	2.7.11.1	mTOR signaling pathway	2.7.11.1
Xenobiotics Biodegradation and Metabolism	Drug metabolism—other enzymes	2.7.1.21; 2.7.1.48; 6.3.5.2	1,4-Dichlorobenzene degradation	3.1.3.1
	Atrazine degradation	3.5.1.5; 1.1.1.205	Benzoate degradation via hydroxylation	2.3.1.9
	Naphthalene and anthracene degradation	1.14.13.1	Biphenyl degradation	4.1.1.77
	Tetrachloroethene degradation	1.1.1.1	Drug metabolism—other enzymes	2.7.1.21
	Benzoate degradation via hydroxylation	4.1.1.44	gamma-Hexachlorocyclohexane degradation	3.8.1.2; 4.2.1.17; 1.14.13.2
	Caprolactam degradation	3.1.1.17	Geraniol degradation	2.3.1.16
	gamma-Hexachlorocyclohexane degradation	3.8.1.2	Styrene degradation	1.13.11.2
	Drug metabolism—other enzymes	3.1.1.1	Toluene and xylene degradation	1.2.1.10
	Geraniol degradation	4.1.3.4	-	-
	Styrene degradation	3.5.1.4	-	-
	Benzoate degradation via hydroxylation	1.13.11.2	-	-
	Metabolism of xenobiotics by cytochrome P450	3.3.2.9	-	-

### Identification of secondary metabolite coding gene clusters

#### Mining of secondary metabolite gene clusters from the whole genome sequences of *Bacillus* strains PBs 12 and PBl 36

**Fig. 5:**
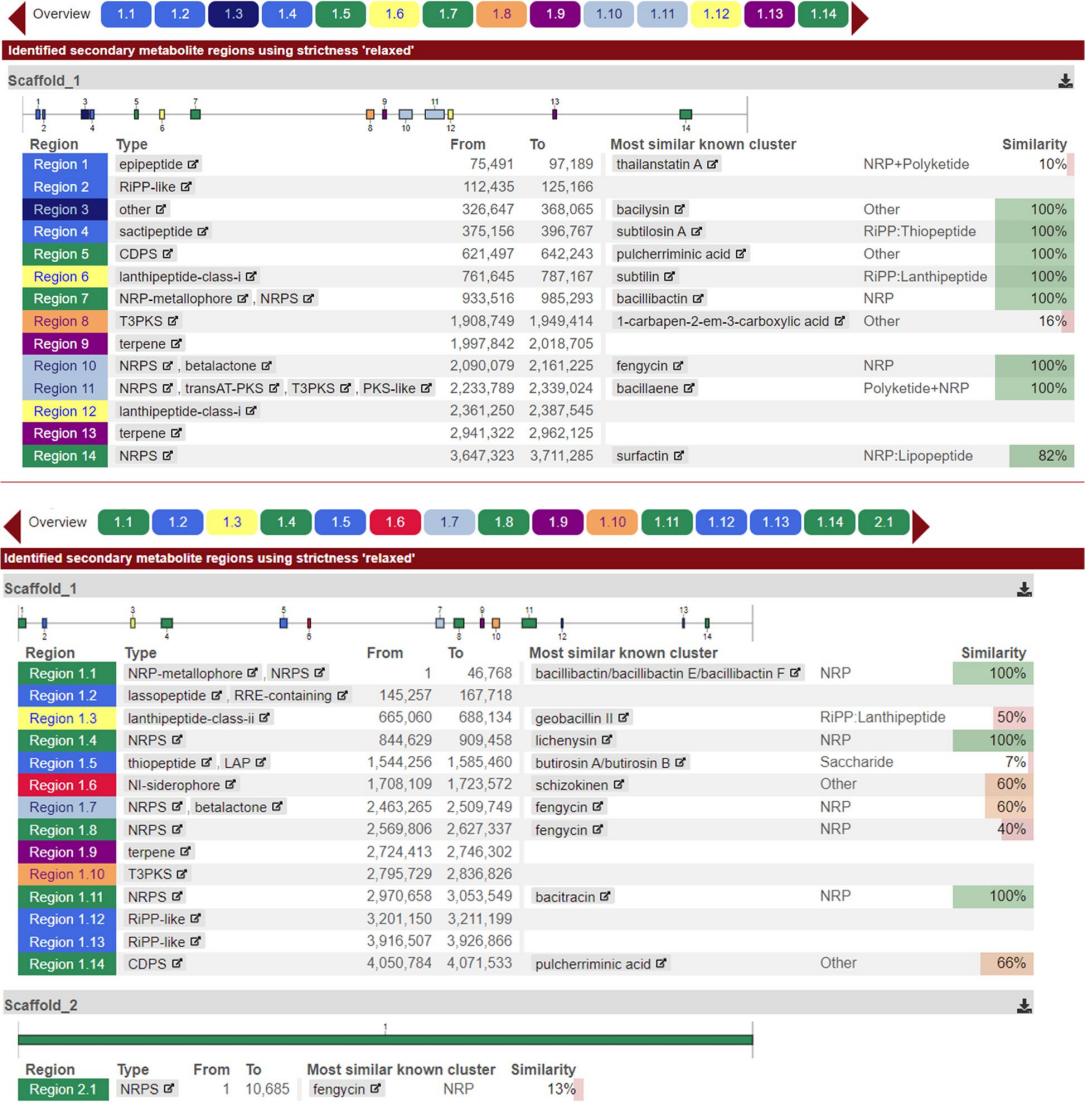
Identification, annotation, and analysis of secondary metabolite regions and related biosynthetic gene clusters in *pearl millet panicle associated Bacillus* species. Note: *Bacillus subtilis* PBs12 has 14 and *Bacillus paralicheniformis* PBl 36 has 15 genomic regions for secondary metabolite synthesis

The antifungal activity exhibited by *Bacillus* strains PBs 12 and PBl 36 was tested by prediction of secondary metabolite coding gene cluster analysis. In total 14 genomic regions in PBs 12 and 15 in PBl 36 identified to be involved in coding for epipeptides, Ripp (Ribosomally synthesized and Post-translationally modified Peptides), sactipeptide, Ripp recognition element (RRE), lanthipeptide, Type III polyketide synthases (PKSs), and NRPS (Non- Ribosomal Peptide Synthetase). Antibiotics coding gene clusters for thailanstatin A, bacilysin, subtilosin, subtilin, bacillibactin, fengycin, surfactin are present in PBs 12 and compounds bacillibactin E/F, geobacillin, lichenysin, butirosin A/B, schizokinen, fengycin, bacitracin are evident in PBl 36 genome (Fig. 5). Further mining of Minimum Information about a Biosynthetic Gene cluster (MiBiG) from known gene clusters revealed PBs 12 and PBl 36 involve in the biosynthesis of a total number of 25,458 and 33,000 secondary metabolite products from each genome. The list of MiBiG products ranged from alkaloids, polyketides, polysaccharides, terpenes, anticoumarins, siderophores, lipo/glycopeptides, antibiotics, etc. (Table 8). The gene clusters with a product count above 100 depict the maximum share of MiBiGs by Alkaloid (7521 and 5341), NRP (4283 and 13328), NRP + Alkaloide (962 and 2753) etc.

**Table 8:** Summary of secondary metabolite gene clusters mined by AntiSmash2.0 in *Bacillus subtilis* PBs12 and *Bacillus paralicheniformis* PBl36

**MiBiG Product**	**Product Count**	**MiBiG Product**	**Product Count**
***Bacillus subtilis* PBs12**		***Bacillus paralicheniformis* PBl36**	
Alkaloid	7521	NRP	13328
Alkaloid + Polyketide:Modular type I	4797	Alkaloid	5341
NRP	4283	Alkaloid + NRP	2753
NRP + Alkaloid	962	Alkaloid + NRP:Lipopeptide	916
NRP + Alkaloid + Polyketide:Iterative type I	646	Alkaloid + NRP + Polyketide:Iterative type I polyketide	850
NRP + Alkaloid + Polyketide:Modular type I	503	Alkaloid + Polyketide	837
NRP + Polyketide	492	Alkaloid + Polyketide:Modular type I polyketide	808
NRP + Polyketide:Enediyne type I	457	Alkaloid + RiPP + Terpene	741
NRP + Polyketide:Iterative type I	396	Alkaloid + Terpene + Saccharide	565
NRP + Polyketide:Modular type I	391	NRP:Beta-lactam	487
NRP + Polyketide:Modular type I + Polyketide:PUFA synthase or related	371	NRP:Beta-lactam + Polyketide:Type II polyketide	475
NRP + Polyketide:Modular type I + Polyketide:Trans-AT type I	327	NRP:Cyclic depsipeptide	435
NRP + Polyketide:Modular type I + Saccharide:Hybrid/tailoring	308	NRP:Cyclic depsipeptide + Polyketide:Iterative type I polyketide	414
NRP + Polyketide:Trans-AT type I	307	NRP:Cyclic depsipeptide + Polyketide:Modular type I polyketide	351
NRP + RiPP	297	NRP:Cyclic depsipeptide + Polyketide:Trans-AT type I polyketide	269
NRP + Saccharide:Hybrid/tailoring	277	NRP:Glycopeptide	255
NRP + Terpene	261	NRP:Glycopeptide + Polyketide:Modular type I polyketide + Saccharide:Hybrid/tailoring saccharide	253
NRP + Terpene + Alkaloid	203	NRP:Glycopeptide + Polyketide:Other polyketide + Saccharide:Hybrid/tailoring saccharide	235
NRP:Beta-lactam	196	NRP:Glycopeptide + Saccharide:Hybrid/tailoring saccharide	227
NRP:Beta-lactam + Polyketide:Modular type I	150	NRP:Lipopeptide	208
NRP:Beta-lactam + Polyketide:Type II	126	NRP:Lipopeptide:Ca + -dependent lipopeptide	176
NRP:Ca + -dependent lipopeptide	114	NRP:Lipopeptide + Polyketide:Iterative type I polyketide	174
NRP:Cyclic depsipeptide	112	NRP:Lipopeptide + Polyketide:Modular type I polyketide	168
NRP:Cyclic depsipeptide + Polyketide:Iterative type I	104	NRP:Lipopeptide + Polyketide:Modular type I polyketide + Saccharide:Hybrid/tailoring saccharide	143
NRP:Cyclic depsipeptide + Polyketide:Modular type I	96	NRP:Lipopeptide + Polyketide:Trans-AT type I polyketide	119
NRP:Cyclic depsipeptide + Polyketide:Trans-AT type I	91	NRP:Lipopeptide + Saccharide:Hybrid/tailoring saccharide	117
NRP:Glycopeptide	85	NRP:NRP siderophore	112
NRP:Glycopeptide + Polyketide:Modular type I + Saccharide:Hybrid/tailoring	84	NRP:NRP siderophore + Polyketide:Modular type I polyketide + Polyketide:Iterative type I polyketide	109
NRP:Glycopeptide + Polyketide:Other + Saccharide:Hybrid/tailoring	83	NRP:Pyrrolobenzodiazepine	92
NRP:Glycopeptide + Saccharide:Hybrid/tailoring	82	NRP:Uridylpeptide + Other:Nucleoside	88
NRP:Lipopeptide	76	NRP + Alkaloid	85
NRP:Lipopeptide + Polyketide:Iterative type I	73	NRP + Alkaloid + Polyketide:Iterative type I polyketide	84
NRP:Lipopeptide + Polyketide:Modular type I	67	NRP + Alkaloid + Polyketide:Modular type I polyketide	79
NRP:Lipopeptide + Polyketide:Modular type I + Saccharide:Hybrid/tailoring	63	NRP + Other	76
NRP:Lipopeptide + Polyketide:Trans-AT type I	53	NRP + Polyketide	73
NRP:Lipopeptide + Saccharide:Hybrid/ tailoring	49	NRP + Polyketide:Enediyne type I polyketide	64
NRP:NRP siderophore	44	NRP + Polyketide:Iterative type I polyketide	62
NRP:NRP siderophore + Polyketide:Modular type I + Polyketide:Iterative type I	42	NRP + Polyketide:Iterative type I polyketide + Polyketide:Enediyne type I polyketide	61
NRP:Uridylpeptide + Other:Nucleoside	31	NRP + Polyketide:Modular type I polyketide	60
Other	30	NRP + Polyketide:Modular type I polyketide + Polyketide:PUFA synthase or related polyketide	55
Other:Aminocoumarin	30	NRP + Polyketide:Modular type I polyketide + Polyketide:Trans-AT type I polyketide	54
Other:Cyclitol	30	NRP + Polyketide:Modular type I polyketide + Saccharide:Hybrid/tailoring saccharide	49
Other:Non-NRP beta-lactam	29	NRP + Polyketide:Trans-AT type I polyketide	47
Other:Nucleoside	29	NRP + Polyketide:Type II polyketide + Saccharide:Hybrid/tailoring saccharide	44
Other:Phenazine	29	NRP + Polyketide + Other	41
Other:Shikimate-derived	29	NRP + Polyketide + Saccharide	35
Polyketide	27	NRP + RiPP	35
Polyketide + NRP	26	NRP + Saccharide	35
Polyketide + NRP + Other:Aminocoumarin	26	NRP + Saccharide:Hybrid/tailoring saccharide	32
Polyketide + NRP + Other:Shikimate-derived	24	NRP + Terpene	32
Polyketide + NRP:Cyclic depsipeptide	24	NRP + Terpene + Alkaloid	30
Polyketide + NRP:Cyclic depsipeptide + Other:Aminocoumarin	23	Other	29
Polyketide + NRP:Lipopeptide	23	Other:Aminocoumarin	28
Polyketide + Other:Aminocoumarin	22	Other:Cyclitol	22
Polyketide + Other:Cyclitol	22	Other:Ectoine	22
Polyketide + Saccharide:Hybrid/tailoring	21	Other:Fatty acid	21
Polyketide + Terpene	18	Other:Non-NRP beta-lactam	21
Polyketide:Iterative type I	17	Other:Non-NRP siderophore	21
Polyketide:Iterative type I + Polyketide:Enediyne type I	17	Other:Nucleoside	21
Polyketide:Iterative type I + Polyketide:Trans-AT type I	16	Other:Phenazine	20
Polyketide:Iterative type I + Polyketide:Type II + Saccharide:Hybrid/tailoring	16	Other:shikimate derived	20
Polyketide:Iterative type I + Saccharide:Hybrid/tailoring	15	Other:Shikimate-derived	20
Polyketide:Modular type I	14	Other + Polyketide	20
Polyketide:Modular type I + Polyketide:Iterative type I + Saccharide:Oligosaccharide	14	Other + Saccharide	19
Polyketide:Modular type I + Polyketide:Trans-AT type I	13	Polyketide	19
Polyketide:Modular type I + Polyketide:Type III	13	Polyketide:Iterative type I polyketide	18
Polyketide:Modular type I + Saccharide:Hybrid/tailoring	13	Polyketide:Iterative type I polyketide + Polyketide:Enediyne type I polyketide	17
Polyketide:Trans-AT type I	13	Polyketide:Iterative type I polyketide + Polyketide:Trans-AT type I polyketide	16
Polyketide:Type II	13	Polyketide:Iterative type I polyketide + Polyketide:Type II polyketide + Saccharide:Hybrid/tailoring saccharide	16
Polyketide:Type II + Polyketide:Type III	13	Polyketide:Iterative type I polyketide + Saccharide:Hybrid/tailoring saccharide	16
Polyketide:Type II + Saccharide:Hybrid/tailoring	12	Polyketide:Modular type I polyketide	15
Polyketide:Type II + Saccharide:Oligosaccharide	11	Polyketide:Modular type I polyketide + Polyketide:Iterative type I polyketide + Saccharide:Oligosaccharide	15
RiPP	11	Polyketide:Modular type I polyketide + Polyketide:Trans-AT type I polyketide	14
RiPP:Bottromycin	10	Polyketide:Modular type I polyketide + Polyketide:Type III polyketide	14
RiPP:Cyanobactin	9	Polyketide:Modular type I polyketide + Saccharide:Hybrid/tailoring saccharide	14
RiPP:Glycocin	9	Polyketide:Trans-AT type I polyketide	13
RiPP:Head-to-tailcyclized peptide	9	Polyketide:Type II polyketide	13
RiPP:Lanthipeptide	9	Polyketide:Type II polyketide + Polyketide:Type III polyketide	13
RiPP:LAP	8	Polyketide:Type II polyketide + Saccharide:Hybrid/tailoring saccharide	13
RiPP:Lassopeptide	7	Polyketide:Type II polyketide + Saccharide:Oligosaccharide	12
RiPP:Linaridin	7	Polyketide + Alkaloid	12
RiPP:Microcin	7	Polyketide + NRP	12
RiPP:Microviridin	7	Polyketide + NRP:Cyclic depsipeptide	12
RiPP:Proteusin	7	Polyketide + NRP:Cyclic depsipeptide + Other:Aminocoumarin	11
RiPP:Sactipeptide	6	Polyketide + NRP:Glycopeptide + Saccharide:Hybrid/tailoring saccharide	11
RiPP:Thiopeptide	5	Polyketide + NRP:Lipopeptide	11
Saccharide	5	Polyketide + NRP + Other	10
Saccharide + NRP	5	Polyketide + NRP + Other:Aminocoumarin	10
Saccharide + NRP:Glycopeptide	5	Polyketide + NRP + Other:Shikimate-derived	10
Saccharide + Polyketide	4	Polyketide + NRP + Saccharide	10
Saccharide + Polyketide:Modular type I + Polyketide:Type II + Other:Aminocoumarin	4	Polyketide + Other	9
Saccharide:Aminoglycoside	4	Polyketide + Other:Aminocoumarin	9
Saccharide:Exopolysaccharide	3	Polyketide + Saccharide	9
Saccharide:Hybrid/tailoring	3	Polyketide + Saccharide:Hybrid/tailoring saccharide	9
Saccharide:Hybrid/tailoring + Other:Aminocoumarin	3	Polyketide + Saccharide:Oligosaccharide	9
Saccharide:Lipopolysaccharide	3	Polyketide + Terpene	9
Saccharide:Oligosaccharide	3	Polyketide + Terpene + Alkaloid	9
Terpene	3	RiPP	9
Terpene + Alkaloid	2	RiPP:Bottromycin	9
Terpene + Polyketide	2	RiPP:Cyanobactin	7
Terpene + Polyketide:Iterative type I	2	RiPP:Glycocin	7
Terpene + Polyketide:Type III	1	RiPP:Head-to-tailcyclized peptide	6
Terpene + RiPP:Glycocin	1	RiPP:Lanthipeptide	6
Terpene + Saccharide	1	RiPP:LAP	6
Terpene + Saccharide:Hybrid/tailoring	1	RiPP:Lassopeptide	6
		RiPP:Linaridin	5
		RiPP:Microcin	5
		RiPP:Microviridin	5
		RiPP:Other	5
		RiPP:Proteusin	5
		RiPP:Sactipeptide	4
		RiPP:Thiopeptide	4
		RiPP + Alkaloid	4
		Saccharide	4
		Saccharide:Aminoglycoside	3
		Saccharide:Hybrid/tailoring saccharide	3
		Saccharide:Hybrid/tailoring saccharide + Other:Aminocoumarin	3
		Saccharide:Hybrid/tailoring saccharide + Other:Nucleoside	2
		Saccharide:Lipopolysaccharide	2
		Saccharide:Oligosaccharide	2
		Saccharide + NRP	2
		Saccharide + NRP:Glycopeptide	2
		Saccharide + Other:Cyclitol	1
		Saccharide + Polyketide	1
		Saccharide + Polyketide:Modular type I polyketide + Polyketide:Type II polyketide + Other:Aminocoumarin	1
		Terpene	1
		Terpene + Alkaloid	1
		Terpene + NRP	1
		Terpene + Polyketide:Iterative type I polyketide	1
		Terpene + Polyketide:Type III polyketide	1
		Terpene + RiPP:Glycocin	1
		Terpene + Saccharide	1
		Terpene + Saccharide:Hybrid/tailoring saccharide	1
**Total**	**25458**		**33000**

## Discussion

Microbes provide a wide range of services and benefits to plants, and in return, they receive reduced carbon and other metabolites [43] for their survival. These microbial communities have been associated with almost all terrestrial plants since their earliest evolution to assist early land plants faced with challenges such as access to nutrients, novel and often stressful conditions, and pathogens, but little is known about their complete functional role [44]. In the present study, pearl millet panicle-associated *Bacillus subtilis* PBs 12 and *Bacillus paralicheniformis* PBl 36 were found to have good plant growth promotion and significant antifungal potential. The two strains were also observed to be the most abundant microbiome member species of pearl millet indicating their versatile roles. *Bacillus* species are one of the most abundant species on plants due to their ability to survive for long periods under harsh environmental conditions. Previously, few studies focused on the pearl millet (leaves, stems, and roots) associated microbiota and its relation to plant growth and stress responses, especially concerning drought and salinity [16, 45–48]. Still, the pearl millet panicle microbiome is under-explored for its potential against foliar diseases including blast, a fungal disease caused by *Magnaporthe grisea* which is a known threat for breeding and cultivation. In this scenario, pearl millet panicle-associated *Bacillus subtilis* PBs 12 was observed as one of the most abundant species on panicles, which produced round to irregular, flat, non-fluid-type, creamy white colonies with irregular borders. The PBs 12 is a gram-positive, endospore-forming bacterium able to utilize citrate, cellulose, and starch. It is also able to produce siderophores, which are known for their elicitor activity to induce plant defense and enhance plant growth promotion [49, 50]. The second prominent isolate, *Bacillus paralicheniformis* PBl 36 produced creamy white rough lichen-like wrinkled colonies with an irregular margin and found motile, gram-positive bacteria with cellulase and starch hydrolysis activity. The morpho-biochemical characters were identical to those of previous characterization studies [51–53]. In 16S rRNA-based phylogenetic analysis, *Bacillus subtilis* PBs 12 (OL674150) showed close clustering with the plant-beneficial bacterial strains *Bacillus subtilis* KA9 (MT491101.1) and *Bacillus subtilis* IB22 (MT590663.1), and *Bacillus licheniformis* PBl 36 (OL674149) showed close clustering with the isolate *Bacillus licheniformis* PZ54 (MT184872.1), a beneficial seed endophyte of wheat expected in vertical transmission from Peninsular India (https://www.ncbi.nlm.nih.gov/nuccore/MT184872.1/). However, whole genome analysis later confirmed that PBl 36 was a member of the *B. paralicheniformis* species. The 16S rRNA, though a universal housekeeping genetic marker to establish bacterial identity, has limitations in providing accuracy at the species level [54, 55]. However, up to the genus level, it will provide a considerable identity for most of the bacteria. After initial characterization, both the strains PBs 12 and PBl 36 were used for further studies.

*Bacillus* species are well known for their plant growth promotion potential by positively regulating the plant metabolism for enhanced growth. To test this behavior, seed priming assays were conducted on pearl millet cv. 7042S with both PBs 12 and PBl 36. The significant shoot and root growth increase along with seedling vigour index in 7-day-old seedlings by PBs 12 and PBl 36 gave hints on their ability to promote plant growth, which can be considered as mutually beneficial plant–microbe interaction. There are several reports on the plant growth promotion activity of *Bacillus subtilis* [22, 26, 56] and *Bacillus paralicheniformis* [57–59]. The direct effects of microbe-induced plant growth are mainly derived either from their capacity to improve the nutritional status of plants or from the production of phytohormones [60]. Several growth-promoting traits, such as indole-3 acetic acid production, zeatin synthesis, phosphate solubilization, and siderophore production, are exhibited by *Bacillus* sp. [59]. Unlike *Bacillus paralicheniformis*, *Bacillus subtilis* is one of the most widely used and studied PGPR (Plant Growth Promoting Rhizobacteria) and a highly promising candidate for agricultural applications [61, 62]. Identification of native pearl millet strains with plant growth promotion abilities will help sustain pearl millet production in near future.

Further, in dual culture direct inhibition assays with different potential fungal plant pathogens (*Sclerotium rolfsii; Magnaporthe grisea; Fusarium solani; Alternaria alternata; Ganoderma* sp. 1 and 2) of Basidiomycota and Ascomycota, including the most economically important pearl millet blast pathogen *Magnaporthe grisea*, the direct inhibition of mycelial growth of all the test pathogens by PBs 12 (60.40–78.70%) and PBl 36 (25.36–69.54%) confirmed their broad-spectrum antifungal activity. Here, PBs-12 has shown a comparatively high inhibitory effect against all pathogens. In our recent study, the leaf endophytic *Bacillus* sp. showed great antifungal activity and induced systemic resistance against rice blast disease caused by *Magnaporthe oryzae* [24]. However, the metabolites that induced such a phenomenon are yet to be explored. The information behind this significant antifungal antibiosis of *Bacillus* sp. can aid in the identification of beneficial bioproducts such as elicitor compounds to stimulate plant immunity against blast disease [63–65]. The ability of bacteria to secrete secondary metabolites such as antibiotics, bacterial organic volatile compounds, cell-wall-degrading enzymes, hormones, plant defense-modulating elicitor compounds, and antioxidants is indeed attributed to their antimicrobial potential [26, 66–69]. To further understand and explore these traits, whole genome sequencing has been performed for both *Bacillus* strains PBs12 and PBl36, and secondary metabolite biosynthetic potential has been studied.

In the present investigation, we have reported the genomes of pearl millet panicle-associated *Bacillus subtilis* PBs 12 and *Bacillus paralicheniformis* PBl 36. *Bacillus subtilis* PBs 12 has a 4020447 bp circular chromosome that consists of 4171 genes with 43.68% G + C content, while *Bacillus paralicheniformis* PBl 36 consists of a 4339481 bp circular chromosome that consists of 4606 genes with 45.83% G + C content. The genome size and GC content of both strains are in accordance with the genomes of the previously sequenced *Bacillus subtilis* [70–72] and *Bacillus paralicheniformis* strains [59, 73–75]. The ANI-based phylogenetic analysis further confirmed the 98.71–98.75% identity of PBs12 with other *Bacillus subtilis* strains, and the *Bacillus subtilis* PBs 12 closest match was strain NCIB—3610 (PRJNA244741) in both orthoANI (99.99) and originalANI (99.99) which was isolated from the USA. Similarly, based on 16 S rRNA, PBl 36 was identified as *B. licheniformis* however, in whole genome-based ANI, it was closely clustered with other *B. paralicheniformis* with 94.37–99.25% identity, and the closest match was *B. paralicheniformis* strain A4-3 (PRJNA552192) isolated from Tomato in South Korea in orthoANI (99.34) and original ANI (99.25). *Bacillus paralicheniformis* has a close evolutionary relationship with *B. licheniformis* and *B. sorensis* [76]. This creates difficulty in the correct identification of *Bacillus paralicheniformis* using a 16S rRNA-based approach.

Further, in PATRIC-based functional annotation, a total of 136 (PBs 12) and 134 (PBl 36) pathway classes were found, where the majority of genes were found to encode the biosynthesis of secondary metabolites (23) followed by xenobiotic biodegradation and metabolism (21), carbohydrate metabolism (15), lipid metabolism (14), amino acid metabolism (13), and metabolism of cofactors and vitamins (12). Two pathways were found to relate to signal transduction, one with the immune system and the other with various metabolic activities. Interestingly, the ability to code ABC-type Fe3 + -siderophore transport system, periplasmic iron-binding component which was evident from PBs 12 genome backed its siderophore production in vitro and indirectly siderophore mediated growth promotion in pearl millet that was observed in seed priming assays [77]. Similarly, trans zeatin ribose (ZR) synthesis by zeatin biosynthesis pathway found in PBl 36 further backed cytokinin mediated growth promotion observed in pearl millet [78]. Due to their ability to synthesize several enzymes and antimicrobial compounds, *B. subtilis* and *B. paralicheniformis* have been used for decades in the biotechnology industry to manufacture enzymes, antibiotics, biochemicals, and several other consumer products [79–82]. Also, they are significant contributors in agriculture, industry, biomaterials, and medicine [82–85]. Previous discoveries of novel metabolites secreted by *Bacillus* sp. such as Bacitracin, Fengycin, Lichenysin and Lantipeptide, siderophores, and cyclic lipopeptides, are evident from genome research studies that help identify biosynthesis pathways and genes involved in the production of various secondary metabolites [83, 85–87]. Identification of key cellular pathways for secondary metabolite biosynthesis helps in the selection of efficient biopesticides and biostimulants for crop protection.

In the prediction of secondary metabolite coding gene clusters, a total of 14 genomic regions in PBs 12 and 15 in PBl 36 identified to be involved in coding for epipeptides, Ripp (Ribosomally synthesized and post-translationally modified Peptides), sactipeptide, Ripp recognition element (RRE), lanthipeptide, Type III polyketide synthases (PKSs), and NRPS (Non- Ribosomal Peptide Synthetase). Several antibiotic coding gene clusters for thailanstatin A, bacilysin, subtilosin, subtilin, bacillibactin, fengycin, surfactin are present in PBs 12 and compounds bacillibactin E/F, geobacillin, lichenysin, butirosin A/B, schizokinen, fengycin, bacitracin are evident in PBl 36 genome. The presence of four antimicrobial Peptide genes, *spaS* (375 bp), *spoVG* (226 bp*)*, *bacA* (498 bp) and *srfAA* (201 bp) in PBs 12 and PBl 36 also further confirmed their potential in the secretion of antimicrobial cyclic lipopeptides such as subtilin, bacylisin and surfactin. The ability of these antimicrobial compounds in plant disease control is enormous, and they are known to initiate a distinct pattern of defense responses in plants. In present study the observed antifungal activity of both the isolates against taxonomically different plant pathogens is mainly attributed to the antimicrobial cyclic lipopeptides production ability observed and validated from their genomes. This is evident when *Bacillus subtilis* PBs 12, shown the presence of subtilin, bacylisin, and surfactin biosynthesis genes, also significantly inhibited all the test plant pathogens, irrespective of their taxonomic position and host plant in dual culture confrontation assays. Previously, mycosubtilin, a cyclic lipopeptide produced from *Bacillus subtilis* shown to induce local resistance against a necrotrophic pathogen, *Botrytis cinerea* [76]. Iturin is another strong antifungal compound secreted by several *Bacillus* species and is well known for its potential to trigger immune responses in various hosts and also for its use in plant disease management, including tomato grey mould, bacterial diseases of cucurbits, tomato late blight, and pepper anthracnose [88–90]. The enhanced levels of subtilin produced by *Bacillus amyloliquefaciens* in wheat spikes are directly linked to the biological control of Fusarium head blight disease caused by *Fusarium graminearum* [91].

Further mining of the Minimum Information about a Biosynthetic Gene Cluster (MiBiG) from known gene clusters revealed that PBs 12 and PBl 36 are involved in the biosynthesis of a total of 25458 and 33000 secondary metabolite products from each genome. The list of MiBiG products ranged from alkaloids, polyketides, polysaccharides, terpenes, anti-coumarins, siderophores, lipo/glycopeptides, antibiotics, etc. *Bacillus,* being an important genus, has been in scientific research and demonstrated its unique abilities for agriculture [92]. *B. subtilis* has revolutionized the industry of bioinoculants, especially biofertilizers [93–95] and biopesticides [96–98]. *B. paralicheniformis* is also known for its nematicidal [99], antifungal [59], antibacterial [100], and growth promotion [59] activities. With this, PBs 12 and PBl 36 isolates inevitably show antifungal potential, but they are unique in that they stand against pearl millet blast disease and other important tree diseases (Malabar neem wilt, Acacia, and Pongamia root rot). This study provided more insights into the genomes of PBs 12 and PBl 36 to further understand their genetic potential. The available genetic information strongly suggests that *Bacillus subtilis* PBs12 and *Bacillus paralicheniformis* PBl 36 have a huge potential to initiate studies on the development of bio-fungicide formulations for both agricultural and forestry pathogens.

## Conclusion

The study showed that *Bacillus subtilis* PBs 12 and *Bacillus paralicheniformis* PBl 36 have the potential to enhance the growth of pearl millet and protect it from the *Magnaporthe grisea* that causes pearl millet blast disease. Also, they have antagonism against taxonomically diverse crop and tree pathogens. This research revealed the genome potential of PBs 12 and PBl 36 which can naturally produce several antimicrobial compounds like siderophores, polysaccharides, antibiotics, and lipopeptides. The findings open up opportunities for further investigation into the pearl millet microbiome, its impact on crop growth and health, and the possibility of developing a biological disease management strategy for crop protection.

### Supplementary Information

**Supplementary Material 1.**

## Data Availability

The whole genome sequence data for *Bacillus subtilis* PBs12 and *Bacillus paralicheniformis* PBl36 genome sequences were submitted to NCBI GenBank (https://www.ncbi.nlm.nih.gov) under the bioproject ID—PRJNA891890 and PRJNA897098 with accession number CP110213 and JAPEZR000000000 respectively.
