# Correction: Comprehensive genomic analysis of *Bacillus subtilis* and *Bacillus paralicheniformis* associated with the pearl millet panicle reveals their antimicrobial potential against important plant pathogens

**DOI:** 10.1186/s12870-024-04975-z

**Published:** 2024-04-10

**Authors:** Mushineni Ashajyothi, Shivannegowda Mahadevakumar, Y. N. Venkatesh, Pullabhotla V. S. R. N. Sarma, Chalasani Danteswari, Alexander Balamurugan, Ganesan Prakash, Vikas Khandelwal, C. Tarasatyavathi, Appa Rao Podile, Kirankumar S. Mysore, Siddaiah Chandranayaka

**Affiliations:** 1 Plant Protection Lab, ICAR-Central Agroforestry Research Institute, Jhansi, Uttar Pradesh 284003 India; 2 Botanical Survey of India, Andaman and Nicobar Regional Centre, Haddo, Port Blair, Andaman and Nicobar Islands 744102 India; 3 Department of Plant Sciences, School of Life Sciences, University of Hyderabad, Hyderabad, Telangana 500046 India; 4 ICAR-Indian Agricultural Research Institute, New Delhi, 110012 India; 5 All India Coordinated Research Project On Pearl Millet, Agriculture University, Jodhpur, Rajasthan 342304 India; 6 Department of Biochemistry and Molecular Biology, Oklahoma State University, Stillwater, OK USA; 7 Department of Studies in Biotechnology, University of Mysore, Mysore, Karnataka 570 006 India

**Correction: BMC Plant Biol 24, 197 (2024)**

10.1186/s12870-024-04881-4

Following publication of the original article [1], the authors identified an error in the author name of Kirankumar S. Mysore.

The incorrect author name is: Mysore S. Kirankumar

The correct author name is: Kirankumar S. Mysore

The author group has been updated above and the original article [1] has been corrected.
