# Supplementary material for: Comprehensive genomic analysis of Bacillus subtilis and Bacillus paralicheniformis associated with the pearl millet panicle reveals their antimicrobial potential against important plant pathogens
Source: BMC Plant Biol. 2024 Mar 18;24:197. doi: 10.1186/s12870-024-04881-4 (PMC10946124; doi:10.1186/s12870-024-04881-4)
Supplement: Supplementary file 1 — Supplementary Material 1. [file 12870_2024_4881_MOESM1_ESM.docx]

**Supplementary Table 1. Biochemical characters of the pearl millet panicle associated and *Bacillus* sp.**

| Characters | Pearl millet associated *Bacillus* strains | |
| --- | --- | --- |
| Organism | *Bacillus licheniformis* | *Bacillus subtilis* |
| Strain | PBl 36 | PBs 12 |
| Location | Mysore, Karnataka, India | Mysore, Karnataka, India |
| Isolation from cultivar | 7042S | 7042S |
| Growth at 37^o^C temperature | (+) | (+) |
| Yellow pigment production on Nutrient agar | (-) | (-) |
| Extracellular polysaccharide production on 1% TZC amended NA medium | (+) | (+) |
| Fluorescence on KB medium | (-) | (-) |
| Rifamycin resistance | (-) | (-) |
| Citrate utilisation | (-) | (+) |
| Lipase and Chitinase activity | (-) | (-) |
| Cellulase activity | (+) | (+) |
| Starch hydrolysis | (+) | (+) |
| Siderophore production | (-) | (+) |
| Indole production | (-) | (-) |
| Ammonia production | (-) | (-) |
| Potassium solubilization | (-) | (-) |
| String formation in 3% KOH | (-) | (-) |
| Gram staining indicated Gram (+/-) | (+) | (+) |

(+) - Positive; (-) - Negative

**[Note:** All the biochemical tests conducted as per the standard protocols and repeated twice before confirming the results**]**

**Supplementary Table 2. Comparative Average Nucleotide Identity analysis for *Bacillus* strains PBs12 and PBl 36**

| **Genome 1** | **Genome 2** | **OrthoANI value (%)** | **Orginal ANI value (%)** |
| --- | --- | --- | --- |
| *Bacillus subtilis PBs12* | *B. subtilis NCIB 3610* | 98.74 | 98.74 |
| *Bacillus subtilis PBs12* | *B. subtilis NBRC13719* | 98.77 | 98.75 |
| *Bacillus subtilis PBs12* | *B. subtilis KCTC 3135* | 98.75 | 98.74 |
| *Bacillus subtilis PBs12* | *B. subtilis DSM 10* | 98.73 | 98.70 |
| *Bacillus subtilis PBs12* | *B. subtilis 168* | 98.78 | 98.71 |
| *Bacillus subtilis PBs12* | *B. halotolerans ZB201702* | 87.19 | 87.01 |
| *Bacillus subtilis PBs12* | *B. velezensis JS25R* | 76.83 | 76.18 |
| *Bacillus subtilis PBs12* | *B. amyloliquefaciens GKT04* | 76.84 | 76.20 |
| *B. subtilis NCIB 3610* | *B. subtilis NBRC13719* | 100.00 | 100.00 |
| *B. subtilis NCIB 3610* | *B. subtilis KCTC 3135* | 100.00 | 99.99 |
| *B. subtilis NCIB 3610* | *B. subtilis DSM 10* | 99.99 | 100.00 |
| *B. subtilis NCIB 3610* | *B. subtilis 168* | 99.99 | 99.99 |
| *B. subtilis NCIB 3610* | *B. halotolerans ZB201702* | 87.33 | 87.12 |
| *B. subtilis NCIB 3610* | *B. velezensis JS25R* | 77.03 | 76.38 |
| *B. subtilis NCIB 3610* | *B. amyloliquefaciens GKT04* | 77.03 | 76.38 |
| *B. subtilis NBRC13719* | *B. subtilis KCTC 3135* | 100.00 | 99.99 |
| *B. subtilis NBRC13719* | *B. subtilis DSM 10* | 99.99 | 100.00 |
| *B. subtilis NBRC13719* | *B. subtilis 168* | 99.99 | 99.99 |
| *B. subtilis NBRC13719* | *B. halotolerans ZB201702* | 87.29 | 87.13 |
| *B. subtilis NBRC13719* | *B. velezensis JS25R* | 76.99 | 76.37 |
| *B. subtilis NBRC13719* | *B. amyloliquefaciens GKT04* | 77.09 | 76.37 |
| *B. subtilis KCTC 3135* | *B. subtilis DSM 10* | 100.00 | 99.99 |
| *B. subtilis KCTC 3135* | *B. subtilis 168* | 99.99 | 99.99 |
| *B. subtilis KCTC 3135* | *B. halotolerans ZB201702* | 87.32 | 87.12 |
| *B. subtilis KCTC 3135* | *B. velezensis JS25R* | 76.95 | 76.37 |
| *B. subtilis KCTC 3135* | *B. amyloliquefaciens GKT04* | 77.17 | 76.37 |
| *B. subtilis DSM 10* | *B. subtilis 168* | 99.99 | 99.99 |
| *B. subtilis DSM 10* | *B. halotolerans ZB201702* | 87.32 | 87.12 |
| *B. subtilis DSM 10* | *B. velezensis JS25R* | 77.05 | 76.40 |
| *B. subtilis DSM 10* | *B. amyloliquefaciens GKT04* | 77.06 | 76.39 |
| *B. subtilis 168* | *B. halotolerans ZB201702* | 87.29 | 87.12 |
| *B. subtilis 168* | *B. velezensis JS25R* | 77.16 | 76.39 |
| *B. subtilis 168* | *B. amyloliquefaciens GKT04* | 76.94 | 76.37 |
| *B. halotolerans ZB201702* | *B. velezensis JS25R* | 77.47 | 76.58 |
| *B. halotolerans ZB201702* | *B. amyloliquefaciens GKT04* | 77.07 | 76.66 |
| *B. velezensis JS25R* | *B. amyloliquefaciens GKT04* | 98.29 | 98.19 |
| *B. paralicheniformis PBl 36* | *B. paralicheniformis PRO109* | 99.12 | 98.99 |
| *B. paralicheniformis PBl 36* | *B. paralicheniformis A4-3* | 99.34 | 99.25 |
| *B. paralicheniformis PBl 36* | *B. paralicheniformis Bac84* | 98.28 | 98.17 |
| *B. paralicheniformis PBl 36* | *B. licheniformis MDJK 30* | 99.05 | 98.95 |
| *B. paralicheniformis PBl 36* | *B. licheniformis ATCC 14580* | 94.63 | 94.37 |
| *B. paralicheniformis PBl 36* | *B. amyloliquefaciens GKT04* | 72.34 | 71.80 |
| *B. paralicheniformis PBl 36* | *B. pumilus Ha06YP001* | 70.12 | 69.23 |
| *B. paralicheniformis PBl 36* | *B. thuringiensis ATCC 10792* | 66.14 | 65.41 |
| *B. paralicheniformis PRO109* | *B. paralicheniformis A4-3* | 99.15 | 98.97 |
| *B. paralicheniformis PRO109* | *B. paralicheniformis Bac84* | 98.38 | 98.32 |
| *B. paralicheniformis PRO109* | *B. licheniformis MDJK 30* | 99.01 | 98.98 |
| *B. paralicheniformis PRO109* | *B. licheniformis ATCC 14580* | 94.47 | 94.30 |
| *B. paralicheniformis PRO109* | *B. amyloliquefaciens GKT04* | 72.60 | 72.01 |
| *B. paralicheniformis PRO109* | *B. pumilus Ha06YP001* | 70.04 | 69.45 |
| *B. paralicheniformis PRO109* | *B. thuringiensis ATCC 10792* | 66.06 | 65.72 |
| *B. paralicheniformis A4-3* | *B. paralicheniformis Bac84* | 98.32 | 98.20 |
| *B. paralicheniformis A4-3* | *B. licheniformis MDJK 30* | 99.12 | 99.01 |
| *B. paralicheniformis A4-3* | *B. licheniformis ATCC 14580* | 94.63 | 94.40 |
| *B. paralicheniformis A4-3* | *B. amyloliquefaciens GKT04* | 72.57 | 71.98 |
| *B. paralicheniformis A4-3* | *B. pumilus Ha06YP001* | 70.12 | 69.45 |
| *B. paralicheniformis A4-3* | *B. thuringiensis ATCC 10792* | 66.08 | 65.77 |
| *B. paralicheniformis Bac84* | *B. licheniformis MDJK 30* | 98.27 | 98.21 |
| *B. paralicheniformis Bac84* | *B. licheniformis ATCC 14580* | 94.96 | 94.72 |
| *B. paralicheniformis Bac84* | *B. amyloliquefaciens GKT04* | 72.63 | 71.93 |
| *B. paralicheniformis Bac84* | *B. pumilus Ha06YP001* | 69.99 | 69.38 |
| *B. paralicheniformis Bac84* | *B. thuringiensis ATCC 10792* | 66.22 | 65.68 |
| *B. licheniformis MDJK 30* | *B. licheniformis ATCC 14580* | 94.67 | 94.44 |
| *B. licheniformis MDJK 30* | *B. amyloliquefaciens GKT04* | 72.71 | 71.99 |
| *B. licheniformis MDJK 30* | *B. pumilus Ha06YP001* | 70.17 | 69.45 |
| *B. licheniformis MDJK 30* | *B. thuringiensis ATCC 10792* | 66.16 | 65.82 |
| *B. licheniformis ATCC 14580* | *B. amyloliquefaciens GKT04* | 72.65 | 71.94 |
| *B. licheniformis ATCC 14580* | *B. pumilus Ha06YP001* | 70.05 | 69.41 |
| *B. licheniformis ATCC 14580* | *B. thuringiensis ATCC 10792* | 66.10 | 65.70 |
| *B. amyloliquefaciens GKT04* | *B. pumilus Ha06YP001* | 70.23 | 69.66 |
| *B. amyloliquefaciens GKT04* | *B. thuringiensis ATCC 10792* | 66.35 | 65.95 |
| *B. pumilus Ha06YP001* | *B. thuringiensis ATCC 10792* | 67.21 | 66.54 |

**Supplementary Table 3. Pathway summary for the *Bacillus* sp.**

| *Bacillus subtilis* PBs12 | *Bacillus paralicheniformis* PBl36 |
| --- | --- |
| Arginine and proline metabolism | Alanine, aspartate and glutamate metabolism |
| Lysine degradation | Arginine and proline metabolism |
| Lysine biosynthesis | Cysteine and methionine metabolism |
| Tryptophan metabolism | Glycine, serine and threonine metabolism |
| Histidine metabolism | Histidine metabolism |
| Tyrosine metabolism | Lysine biosynthesis |
| Cysteine and methionine metabolism | Lysine degradation |
| Valine, leucine and isoleucine biosynthesis | Phenylalanine metabolism |
| Phenylalanine metabolism | Phenylalanine, tyrosine and tryptophan biosynthesis |
| Phenylalanine, tyrosine and tryptophan biosynthesis | Tryptophan metabolism |
| Glycine, serine and threonine metabolism | Tyrosine metabolism |
| Valine, leucine and isoleucine degradation | Valine, leucine and isoleucine biosynthesis |
| Alanine, aspartate and glutamate metabolism | Valine, leucine and isoleucine degradation |
| Polyketide sugar unit biosynthesis | Biosynthesis of 12-, 14- and 16-membered macrolides |
| Biosynthesis of ansamycins | Biosynthesis of ansamycins |
| Biosynthesis of siderophore group nonribosomal peptides | Biosynthesis of siderophore group nonribosomal peptides |
| Biosynthesis of type II polyketide backbone | Biosynthesis of type II polyketide backbone |
| Biosynthesis of type II polyketide products | Biosynthesis of type II polyketide products |
| Biosynthesis of 12-, 14- and 16-membered macrolides | Polyketide sugar unit biosynthesis |
| Biosynthesis of vancomycin group antibiotics | Anthocyanin biosynthesis |
| Streptomycin biosynthesis | beta-Lactam resistance |
| Brassinosteroid biosynthesis | Betalain biosynthesis |
| Flavone and flavonol biosynthesis | Brassinosteroid biosynthesis |
| Diterpenoid biosynthesis | Caffeine metabolism |
| Stilbenoid, diarylheptanoid and gingerol biosynthesis | Carotenoid biosynthesis |
| Caffeine metabolism | Diterpenoid biosynthesis |
| Betalain biosynthesis | Flavone and flavonol biosynthesis |
| Tetracycline biosynthesis | Flavonoid biosynthesis |
| Isoquinoline alkaloid biosynthesis | Insect hormone biosynthesis |
| Terpenoid backbone biosynthesis | Isoflavonoid biosynthesis |
| Zeatin biosynthesis | Isoquinoline alkaloid biosynthesis |
| Tropane, piperidine and pyridine alkaloid biosynthesis | Limonene and pinene degradation |
| Carotenoid biosynthesis | Novobiocin biosynthesis |
| Puromycin biosynthesis | Penicillin and cephalosporin biosynthesis |
| Novobiocin biosynthesis | Phenylpropanoid biosynthesis |
| Insect hormone biosynthesis | Puromycin biosynthesis |
| Limonene and pinene degradation | Stilbenoid, diarylheptanoid and gingerol biosynthesis |
| Penicillin and cephalosporin biosynthesis | Streptomycin biosynthesis |
| Anthocyanin biosynthesis | Terpenoid backbone biosynthesis |
| Phenylpropanoid biosynthesis | Tetracycline biosynthesis |
| Flavonoid biosynthesis | Tropane, piperidine and pyridine alkaloid biosynthesis |
| Isoflavonoid biosynthesis | Zeatin biosynthesis |
| beta-Lactam resistance | Amino sugar and nucleotide sugar metabolism |
| Glycolysis / Gluconeogenesis | Ascorbate and aldarate metabolism |
| Inositol phosphate metabolism | Butanoate metabolism |
| Glyoxylate and dicarboxylate metabolism | C5-Branched dibasic acid metabolism |
| Pentose and glucuronate interconversions | Citrate cycle (TCA cycle) |
| Amino sugar and nucleotide sugar metabolism | Fructose and mannose metabolism |
| Pyruvate metabolism | Galactose metabolism |
| Pentose phosphate pathway | Glycolysis / Gluconeogenesis |
| Starch and sucrose metabolism | Glyoxylate and dicarboxylate metabolism |
| Citrate cycle (TCA cycle) | Inositol phosphate metabolism |
| Galactose metabolism | Pentose and glucuronate interconversions |
| Butanoate metabolism | Pentose phosphate pathway |
| Propanoate metabolism | Propanoate metabolism |
| C5-Branched dibasic acid metabolism | Pyruvate metabolism |
| Fructose and mannose metabolism | Starch and sucrose metabolism |
| Ascorbate and aldarate metabolism | Carbon fixation in photosynthetic organisms |
| Methane metabolism | Methane metabolism |
| Photosynthesis | Nitrogen metabolism |
| Oxidative phosphorylation | Oxidative phosphorylation |
| Nitrogen metabolism | Photosynthesis |
| Reductive carboxylate cycle (CO2 fixation) | Reductive carboxylate cycle (CO2 fixation) |
| Sulfur metabolism | Sulfur metabolism |
| Carbon fixation in photosynthetic organisms | Glycosaminoglycan degradation |
| Peptidoglycan biosynthesis | Glycosphingolipid biosynthesis - ganglio series |
| Lipopolysaccharide biosynthesis | Glycosphingolipid biosynthesis - globo series |
| Glycosphingolipid biosynthesis - lacto and neolacto series | Glycosphingolipid biosynthesis - lacto and neolacto series |
| O-Glycan biosynthesis | Glycosylphosphatidylinositol(GPI)-anchor biosynthesis |
| Glycosphingolipid biosynthesis - ganglio series | High-mannose type N-glycan biosynthesis |
| Glycosaminoglycan degradation | Lipopolysaccharide biosynthesis |
| Glycosphingolipid biosynthesis - globo series | O-Glycan biosynthesis |
| High-mannose type N-glycan biosynthesis | Peptidoglycan biosynthesis |
| Glycosylphosphatidylinositol(GPI)-anchor biosynthesis | T cell receptor signaling pathway |
| T cell receptor signaling pathway | alpha-Linolenic acid metabolism |
| Primary bile acid biosynthesis | Arachidonic acid metabolism |
| Synthesis and degradation of ketone bodies | Biosynthesis of unsaturated fatty acids |
| Glycerolipid metabolism | C21-Steroid hormone metabolism |
| Arachidonic acid metabolism | Ether lipid metabolism |
| Fatty acid biosynthesis | Fatty acid biosynthesis |
| Fatty acid elongation in mitochondria | Fatty acid elongation in mitochondria |
| Fatty acid metabolism | Fatty acid metabolism |
| Sphingolipid metabolism | Glycerolipid metabolism |
| Glycerophospholipid metabolism | Glycerophospholipid metabolism |
| Linoleic acid metabolism | Linoleic acid metabolism |
| Secondary bile acid biosynthesis | Primary bile acid biosynthesis |
| alpha-Linolenic acid metabolism | Sphingolipid metabolism |
| Ether lipid metabolism | Synthesis and degradation of ketone bodies |
| C21-Steroid hormone metabolism | Biotin metabolism |
| Riboflavin metabolism | Folate biosynthesis |
| Ubiquinone and other terpenoid-quinone biosynthesis | Lipoic acid metabolism |
| Porphyrin and chlorophyll metabolism | Nicotinate and nicotinamide metabolism |
| Thiamine metabolism | One carbon pool by folate |
| Biotin metabolism | Pantothenate and CoA biosynthesis |
| Pantothenate and CoA biosynthesis | Porphyrin and chlorophyll metabolism |
| Nicotinate and nicotinamide metabolism | Retinol metabolism |
| One carbon pool by folate | Riboflavin metabolism |
| Folate biosynthesis | Thiamine metabolism |
| Retinol metabolism | Ubiquinone and other terpenoid-quinone biosynthesis |
| Vitamin B6 metabolism | Vitamin B6 metabolism |
| Lipoic acid metabolism | beta-Alanine metabolism |
| beta-Alanine metabolism | Cyanoamino acid metabolism |
| Taurine and hypotaurine metabolism | D-Alanine metabolism |
| Glutathione metabolism | D-Arginine and D-ornithine metabolism |
| D-Glutamine and D-glutamate metabolism | D-Glutamine and D-glutamate metabolism |
| Selenoamino acid metabolism | Glutathione metabolism |
| Cyanoamino acid metabolism | Phosphonate and phosphinate metabolism |
| D-Alanine metabolism | Selenoamino acid metabolism |
| Phosphonate and phosphinate metabolism | Taurine and hypotaurine metabolism |
| D-Arginine and D-ornithine metabolism | Purine metabolism |
| Pyrimidine metabolism | Pyrimidine metabolism |
| Purine metabolism | mTOR signaling pathway |
| Phosphatidylinositol signaling system | Phosphatidylinositol signaling system |
| mTOR signaling pathway | Aminoacyl-tRNA biosynthesis |
| Aminoacyl-tRNA biosynthesis | 1- and 2-Methylnaphthalene degradation |
| Drug metabolism - other enzymes | 1,1,1-Trichloro-2,2-bis(4-chlorophenyl) ethane (DDT) degradation |
| Atrazine degradation | 1,4-Dichlorobenzene degradation |
| Naphthalene and anthracene degradation | 2,4-Dichlorobenzoate degradation |
| 1,4-Dichlorobenzene degradation | Atrazine degradation |
| Styrene degradation | Benzoate degradation via hydroxylation |
| 1- and 2-Methylnaphthalene degradation | Biphenyl degradation |
| Tetrachloroethene degradation | Bisphenol A degradation |
| Drug metabolism - cytochrome P450 | Caprolactam degradation |
| Metabolism of xenobiotics by cytochrome P450 | Drug metabolism - cytochrome P450 |
| Ethylbenzene degradation | Drug metabolism - other enzymes |
| Benzoate degradation via hydroxylation | Ethylbenzene degradation |
| Toluene and xylene degradation | gamma-Hexachlorocyclohexane degradation |
| Trinitrotoluene degradation | Geraniol degradation |
| Biphenyl degradation | Metabolism of xenobiotics by cytochrome P450 |
| Caprolactam degradation | Naphthalene and anthracene degradation |
| 2,4-Dichlorobenzoate degradation | Styrene degradation |
| gamma-Hexachlorocyclohexane degradation | Tetrachloroethene degradation |
| Geraniol degradation | Toluene and xylene degradation |
| Bisphenol A degradation | Trinitrotoluene degradation |
| 1,1,1-Trichloro-2,2-bis(4-chlorophenyl) ethane (DDT) degradation |  |
| Fluorobenzoate degradation |  |

**Supplementary Table 4. List of subsystems present in *Bacillus* sp. genomes**

| *Bacillus subtilis* PBs12 | *Bacillus paralicheniformis* PBl36 |
| --- | --- |
| 2-oxoglutarate dehydrogenase | 2-oxoglutarate dehydrogenase |
| 5-methylaminomethyl-2-thiouridine | 5-methylaminomethyl-2-thiouridine |
| A Hypothetical Protein Related to Proline Metabolism | A Hypothetical Protein Related to Proline Metabolism |
| Acetoin, butanediol metabolism | Acetoin, butanediol metabolism |
| Acetolactate synthase subunits | Acetolactate synthase subunits |
| Acyl carrier protein | Acyl carrier protein |
| Adenosyl nucleosidases | Adenosyl nucleosidases |
| Alpha-acetolactate operon | Alpha-acetolactate operon |
| Amino acid racemase | Amino acid racemase |
| Aminoglycoside modifying enzymes: O-nucleotidyltransferases | Aminoglycoside modifying enzymes: O-nucleotidyltransferases |
| An Arabinose Sensor | An Arabinose Sensor |
| Anaerobic module of TCA | Anaerobic module of TCA |
| Antibiotic targets in cell wall biosynthesis | Antibiotic targets in cell wall biosynthesis |
| Antibiotic targets in DNA processing | Antibiotic targets in DNA processing |
| Antibiotic targets in metabolic pathways | Antibiotic targets in metabolic pathways |
| Antibiotic targets in protein synthesis | Antibiotic targets in protein synthesis |
| Antibiotic targets in transcription | Antibiotic targets in transcription |
| Arginine biosynthesis | Arginine biosynthesis |
| Arginine decarboxylase and Agmatinase cluster | Arginine decarboxylase and Agmatinase cluster |
| Arsenic resistance | Arsenic resistance |
| Aspartate to Homoserine module | Aspartate to Homoserine module |
| Aspartate to Threonine Module | Aspartate to Threonine Module |
| ATP-dependent Nuclease | ATP-dependent Nuclease |
| ATP-dependent RNA helicases, bacterial | ATP-dependent RNA helicases, bacterial |
| Bacillibactin Siderophore | Bacillibactin Siderophore |
| Bacillithiol synthesis | Bacillithiol synthesis |
| Bacitracin resistance | Bacitracin resistance |
| Bacterial cell division related cluster 2 | Bacterial cell division related cluster 2 |
| Bacterial checkpoint-control-related cluster | Bacterial checkpoint-control-related cluster |
| Barnase-barstar complex | Barnase-barstar complex |
| Beta-lactamases Ambler class A | Beta-lactamases Ambler class A |
| Biogenesis of c-type cytochromes | Biogenesis of c-type cytochromes |
| Biogenesis of cytochrome c oxidases | Biogenesis of cytochrome c oxidases |
| Biotin biosynthesis | Biotin biosynthesis |
| Biotin synthesis & utilization | Biotin synthesis & utilization |
| Biotin synthesis cluster | Biotin synthesis cluster |
| Branched-Chain Amino Acid Biosynthesis | Branched-Chain Amino Acid Biosynthesis |
| Branched-chain amino acids and alpha-keto acids utilization as energy sources | Branched-chain amino acids and alpha-keto acids utilization as energy sources |
| Cadmium resistance | Cadmium resistance |
| Carbon storage regulator | Carbon storage regulator |
| Carbonic anhydrase | Carbonic anhydrase |
| Cardiolipin biosynthesis | Cardiolipin biosynthesis |
| Cell division cluster | Cell division cluster |
| Cell division related cluster | Cell division related cluster |
| Cell division related cluster including coaD | Cell division related cluster including coaD |
| Cell envelope-associated LytR-CpsA-Psr transcriptional attenuators | Cell envelope-associated LytR-CpsA-Psr transcriptional attenuators |
| Chaperones GroEL GroES and Thermosome | Chaperones GroEL GroES and Thermosome |
| Chemotaxis in Escherichia coli and Bacillus subtilis | Chemotaxis in Escherichia coli and Bacillus subtilis |
| Chloramphenicol resistance | Chloramphenicol resistance |
| Choline uptake and conversion to betaine clusters | Choline uptake and conversion to betaine clusters |
| Chorismate Synthesis | Chorismate Synthesis |
| Citrate uptake cluster | Citrate uptake cluster |
| Cluster containing Glutathione synthetase | Cluster containing Glutathione synthetase |
| Coat proteins CotJABC | Coat proteins CotJABC |
| Cob(I)alamin adenosyltransferase | Cob(I)alamin adenosyltransferase |
| Coenzyme A -- gjo | Coenzyme A -- gjo |
| Coenzyme A Biosynthesis cluster | Coenzyme A Biosynthesis cluster |
| Cold shock proteins of CSP family | Cold shock proteins of CSP family |
| Copper Transport System | Copper Transport System |
| Copper uptake system CopCD | Copper uptake system CopCD |
| CtsR and MscAB regulation of protein degradation | CtsR and MscAB regulation of protein degradation |
| Cysteine synthesis | Cysteine synthesis |
| Cytochrome d ubiquinol oxidase operon | Cytochrome d ubiquinol oxidase operon |
| D-alanylation of teichoic acid | D-alanylation of teichoic acid |
| Damaged nicotinamide nucleotide NAD(P)HX repair | Damaged nicotinamide nucleotide NAD(P)HX repair |
| DAP (1,3-diaminopropane) production | DAP (1,3-diaminopropane) production |
| De Novo Pyrimidine Synthesis | De Novo Pyrimidine Synthesis |
| Dehydrogenase complexes | Dehydrogenase complexes |
| DeNovo Purine Biosynthesis | DeNovo Purine Biosynthesis |
| D-Galacturonate and D-Glucuronate Utilization | D-Galacturonate and D-Glucuronate Utilization |
| D-gluconate and ketogluconates metabolism | D-gluconate and ketogluconates metabolism |
| Diaminopimelate Synthesis | Diaminopimelate Synthesis |
| Dipicolinate Synthesis | Dipicolinate Synthesis |
| DNA internalization-related cluster | DNA internalization-related cluster |
| DNA processing cluster | DNA processing cluster |
| DNA Repair Base Excision | DNA Repair Base Excision |
| DNA repair system including RecA, MutS and a hypothetical protein | DNA repair system including RecA, MutS and a hypothetical protein |
| DNA repair, bacterial | DNA repair, bacterial |
| DNA repair, bacterial DinG and relatives | DNA repair, bacterial DinG and relatives |
| DNA repair, bacterial MutHLS system | DNA repair, bacterial MutHLS system |
| DNA repair, bacterial RecBCD pathway | DNA repair, bacterial RecBCD pathway |
| DNA repair, bacterial RecFOR pathway | DNA repair, bacterial RecFOR pathway |
| DNA repair, bacterial SbcCD exonuclease | DNA repair, bacterial SbcCD exonuclease |
| DNA repair, bacterial UvrD and related helicases | DNA repair, bacterial UvrD and related helicases |
| DNA repair, UvrABC system | DNA repair, UvrABC system |
| DNA topoisomerases, Type II, ATP-dependent | DNA topoisomerases, Type II, ATP-dependent |
| Dpp dipeptide ABC transport system | Dpp dipeptide ABC transport system |
| D-tyrosyl-tRNA(Tyr) deacylase | D-tyrosyl-tRNA(Tyr) deacylase |
| EcsAB transporter affecting expression and secretion of secretory preproteins | EcsAB transporter affecting expression and secretion of secretory preproteins |
| Efflux ABC transporters BmrCD and LmrCD involved in multidrug resistance | Efflux ABC transporters BmrCD and LmrCD involved in multidrug resistance |
| Enoyl-[ACP] reductases disambiguation | Enoyl-[ACP] reductases disambiguation |
| Entner-Doudoroff Pathway | Entner-Doudoroff Pathway |
| ESAT-6 protein secretion system in Mycobacteria (locus ESX-5) | ESAT-6 protein secretion system in Mycobacteria (locus ESX-5) |
| ESAT-6 proteins secretion system in Firmicutes and Actinobacteria | ESAT-6 proteins secretion system in Firmicutes and Actinobacteria |
| ESAT-6 proteins secretion system in Firmicutes extended | ESAT-6 proteins secretion system in Firmicutes extended |
| ESAT-6 proteins secretion system in Mycobacteria (locus ESX-1) | ESAT-6 proteins secretion system in Mycobacteria (locus ESX-1) |
| Exosporium | Exosporium |
| F0F1-type ATP synthase | F0F1-type ATP synthase |
| Fatty Acid Biosynthesis cluster | Fatty Acid Biosynthesis cluster |
| Fatty Acid Biosynthesis FASII | Fatty Acid Biosynthesis FASII |
| Fatty acid catabolic operon fadN-fadA-fadE (yusJKL) | Fatty acid catabolic operon fadN-fadA-fadE (yusJKL) |
| Fatty acid synthesis | Fatty acid synthesis |
| Fermentations: Lactate | Fermentations: Lactate |
| Fermentations: Mixed acid | Fermentations: Mixed acid |
| Fe-S cluster assembly | Fe-S cluster assembly |
| Flagellum | Flagellum |
| Folate Biosynthesis | Folate Biosynthesis |
| Folate biosynthesis cluster | Folate biosynthesis cluster |
| Forespore to mother cell channel | Forespore to mother cell channel |
| Formaldehyde assimilation: Ribulose monophosphate pathway | Formaldehyde assimilation: Ribulose monophosphate pathway |
| Fosfomycin resistance | Fosfomycin resistance |
| FtsEX cell-division-associated signaling system | FtsEX cell-division-associated signaling system |
| Fusidic acid resistance | Fusidic acid resistance |
| Galactose utilization | Galactose utilization |
| Glutamate dehydrogenases | Glutamate dehydrogenases |
| Glutamine synthetases | Glutamine synthetases |
| Glutathione: Non-redox reactions | Glutathione: Non-redox reactions |
| Glutathione: Redox cycle | Glutathione: Redox cycle |
| Glycerolipid and Glycerophospholipid Metabolism in Bacteria | Glycerolipid and Glycerophospholipid Metabolism in Bacteria |
| Glycine cleavage system | Glycine cleavage system |
| Glycogen metabolism | Glycogen metabolism |
| Glycolate, glyoxylate interconversions | Glycolate, glyoxylate interconversions |
| Glycolysis and Gluconeogenesis | Glycolysis and Gluconeogenesis |
| Glycyl-tRNA synthetase | Glycyl-tRNA synthetase |
| Glyoxylate bypass | Glyoxylate bypass |
| GMP synthase | GMP synthase |
| Heat shock dnaK gene cluster extended | Heat shock dnaK gene cluster extended |
| Heme and heme d1 biosynthesis from siroheme | Heme and heme d1 biosynthesis from siroheme |
| Heme Biosynthesis: protoporphyrin-, coproporphyrin- and siroheme-dependent pathways | Heme Biosynthesis: protoporphyrin-, coproporphyrin- and siroheme-dependent pathways |
| Heme O and Heme A biosynthesis | Heme O and Heme A biosynthesis |
| Heme O and Heme A biosynthesis (with selected terminal oxidases) | Heme O and Heme A biosynthesis (with selected terminal oxidases) |
| Heme, hemin uptake and utilization systems in GramPositives | Heme, hemin uptake and utilization systems in GramPositives |
| Hfl operon | Hfl operon |
| High affinity phosphate transporter and control of PHO regulon | High affinity phosphate transporter and control of PHO regulon |
| Histidine Biosynthesis -- gjo | Histidine Biosynthesis -- gjo |
| HMG CoA Synthesis | HMG CoA Synthesis |
| HPr catabolite repression system | HPr catabolite repression system |
| Hydrolysis of sphingomyelin | Hydrolysis of sphingomyelin |
| Hydroxyaromatic decarboxylase family | Hydroxyaromatic decarboxylase family |
| IMP and Xanthine conversion to XMP and GMP module | IMP and Xanthine conversion to XMP and GMP module |
| Inositol catabolism | Inositol catabolism |
| Isoprenoid Biosynthesis: Interconversions | Isoprenoid Biosynthesis: Interconversions |
| Lactose utilization | Lactose utilization |
| Late competence | Late competence |
| Lipid orphans | Lipid orphans |
| Lipoic acid metabolism | Lipoic acid metabolism |
| Lipoylated proteins | Lipoylated proteins |
| L-serine dehydratase subunits | L-serine dehydratase subunits |
| Lysine DAP biosynthetic pathway | Lysine DAP biosynthetic pathway |
| Macrolides, lincosamides, streptogramins, ketolides, oxazolidinones (MLSKO) resistance: enzymatic degradation | Macrolides, lincosamides, streptogramins, ketolides, oxazolidinones (MLSKO) resistance: enzymatic degradation |
| Macrolides, lincosamides, streptogramins, ketolides, oxazolidinones (MLSKO) resistance: rRNA methylases | Macrolides, lincosamides, streptogramins, ketolides, oxazolidinones (MLSKO) resistance: rRNA methylases |
| Macromolecular synthesis operon | Macromolecular synthesis operon |
| Magnesium transport | Magnesium transport |
| MazEF toxin-antitoxing (programmed cell death) system | MazEF toxin-antitoxing (programmed cell death) system |
| Menaquinone biosynthesis from chorismate via 1,4-dihydroxy-2-naphthoate | Menaquinone biosynthesis from chorismate via 1,4-dihydroxy-2-naphthoate |
| Metabolite repair | Metabolite repair |
| Metal chelatases | Metal chelatases |
| Methionine Salvage | Methionine Salvage |
| Methylenetetrahydrofolate reductase | Methylenetetrahydrofolate reductase |
| Methylglyoxal Metabolism | Methylglyoxal Metabolism |
| Methylhydantoinase | Methylhydantoinase |
| Methylthiotransferases | Methylthiotransferases |
| Molybdenum cofactor biosynthesis | Molybdenum cofactor biosynthesis |
| Muconate lactonizing enzyme family | Muconate lactonizing enzyme family |
| Multi-subunit cation antiporter | Multi-subunit cation antiporter |
| Mupirocin resistance | Mupirocin resistance |
| Murein hydrolase regulation and cell death | Murein hydrolase regulation and cell death |
| Mycobacterial lipid and multidrug efflux system proteins MmpL, MmpS | Mycobacterial lipid and multidrug efflux system proteins MmpL, MmpS |
| NAD and NADP cofactor biosynthesis bacterial | NAD and NADP cofactor biosynthesis bacterial |
| NAD and NADP cofactor biosynthesis global | NAD and NADP cofactor biosynthesis global |
| NADH ubiquinone oxidoreductase vs. multi-subunit cation antiporter | NADH ubiquinone oxidoreductase vs. multi-subunit cation antiporter |
| NhaA, NhaD and Sodium-dependent phosphate transporters | NhaA, NhaD and Sodium-dependent phosphate transporters |
| Nickel-requiring | Nickel-requiring |
| Nonmevalonate Branch of Isoprenoid Biosynthesis | Nonmevalonate Branch of Isoprenoid Biosynthesis |
| Nucleoside triphosphate pyrophosphohydrolase MazG | Nucleoside triphosphate pyrophosphohydrolase MazG |
| Nucleoside uptake and degradation cluster | Nucleoside uptake and degradation cluster |
| Nudix proteins (nucleoside triphosphate hydrolases) | Nudix proteins (nucleoside triphosphate hydrolases) |
| One-carbon metabolism by tetrahydropterines | One-carbon metabolism by tetrahydropterines |
| Osmoregulation | Osmoregulation |
| Pentose phosphate pathway | Pentose phosphate pathway |
| Peptidase clustering with DAP | Peptidase clustering with DAP |
| Peptide methionine sulfoxide reductase | Peptide methionine sulfoxide reductase |
| Peptidyl-prolyl cis-trans isomerase | Peptidyl-prolyl cis-trans isomerase |
| Periplasmic disulfide interchange | Periplasmic disulfide interchange |
| Phd-Doc, YdcE-YdcD toxin-antitoxin (programmed cell death) systems | Phd-Doc, YdcE-YdcD toxin-antitoxin (programmed cell death) systems |
| Phenylalanine and Tyrosine synthesis 1 | Phenylalanine and Tyrosine synthesis 1 |
| Phr peptides - Rap phosphatases signaling | Phr peptides - Rap phosphatases signaling |
| Polyamine Metabolism | Polyamine Metabolism |
| Poly-gamma-glutamate biosynthesis | Poly-gamma-glutamate biosynthesis |
| Potassium-transporting ATPase | Potassium-transporting ATPase |
| Programmed frameshift | Programmed frameshift |
| Proline biosynthesis (for review) | Proline biosynthesis (for review) |
| Proline Synthesis | Proline Synthesis |
| Proline, 4-hydroxyproline uptake and utilization | Proline, 4-hydroxyproline uptake and utilization |
| Proteasome bacterial | Proteasome bacterial |
| Protection from Reactive Oxygen Species | Protection from Reactive Oxygen Species |
| Protein chaperones | Protein chaperones |
| Protein degradation | Protein degradation |
| Purine nucleotide synthesis regulator | Purine nucleotide synthesis regulator |
| Pyridoxin (Vitamin B6) Biosynthesis | Pyridoxin (Vitamin B6) Biosynthesis |
| Pyruvate Alanine Serine Interconversions | Pyruvate Alanine Serine Interconversions |
| Pyruvate metabolism I: anaplerotic reactions, PEP | Pyruvate metabolism I: anaplerotic reactions, PEP |
| Pyruvate metabolism II: acetyl-CoA, acetogenesis from pyruvate | Pyruvate metabolism II: acetyl-CoA, acetogenesis from pyruvate |
| Queuosine and archaeosine | Queuosine and archaeosine |
| Quinone oxidoreductase family | Quinone oxidoreductase family |
| RecA and RecX | RecA and RecX |
| Repair of Iron Centers | Repair of Iron Centers |
| Replication-associated recombination protein RarA | Replication-associated recombination protein RarA |
| Resistance to chromium compounds | Resistance to chromium compounds |
| Resistance to Daptomycin | Resistance to Daptomycin |
| Resistance to Triclosan | Resistance to Triclosan |
| Riboflavin, FMN and FAD metabolism with fusion events | Riboflavin, FMN and FAD metabolism with fusion events |
| Ribonuclease H | Ribonuclease H |
| Ribonuclease J family | Ribonuclease J family |
| Ribonucleotide reduction | Ribonucleotide reduction |
| Ribosomal protein S5p acylation | Ribosomal protein S5p acylation |
| Ribosomal proteins, single-copy | Ribosomal proteins, single-copy |
| Ribosomal proteins, zinc requirement | Ribosomal proteins, zinc requirement |
| Ribosome activity modulation | Ribosome activity modulation |
| Ribosome LSU, bacterial | Ribosome LSU, bacterial |
| RNA polymerase, bacterial | RNA polymerase, bacterial |
| RNA processing and degradation, bacterial | RNA processing and degradation, bacterial |
| RNA pseudouridine synthases | RNA pseudouridine synthases |
| RuvABC plus a hypothetical | RuvABC plus a hypothetical |
| S-Adenosyl-L-homocysteine recycling | S-Adenosyl-L-homocysteine recycling |
| Siderophore Anthrachelin | Siderophore Anthrachelin |
| Siderophore assembly kit | Siderophore assembly kit |
| Signal peptidase | Signal peptidase |
| S-methylmethionine | S-methylmethionine |
| Spore germinant receptors | Spore germinant receptors |
| Spore germination | Spore germination |
| Sporulation Cluster | Sporulation Cluster |
| Sporulation Cluster III A | Sporulation Cluster III A |
| Sporulation gene orphans | Sporulation gene orphans |
| Sporulation proteins SigEG cluster | Sporulation proteins SigEG cluster |
| Sporulation proteins SpoIIIAA-SpoIIIAH | Sporulation proteins SpoIIIAA-SpoIIIAH |
| Sporulation proteins SpoVA cluster | Sporulation proteins SpoVA cluster |
| Sporulation-associated proteins with broader functions | Sporulation-associated proteins with broader functions |
| SpoVS protein family | SpoVS protein family |
| Stress proteins YciF, YciE | Stress proteins YciF, YciE |
| Stringent Response, (p)ppGpp metabolism | Stringent Response, (p)ppGpp metabolism |
| Succinate dehydrogenase and Fumarate reductase cpmlexes | Succinate dehydrogenase and Fumarate reductase cpmlexes |
| Sucrose to levan conversions | Sucrose to levan conversions |
| TCA Cycle | TCA Cycle |
| Tetracycline resistance, all mechanisms | Tetracycline resistance, all mechanisms |
| Thiamin, hydroxymethylpyrimidine selected transporters | Thiamin, hydroxymethylpyrimidine selected transporters |
| Thiamin, thiazole, hydroxymethylpyrimidine salvage and uptake | Thiamin, thiazole, hydroxymethylpyrimidine salvage and uptake |
| Thiazole.oxazole-modified microcins | Thiazole.oxazole-modified microcins |
| Threonine synthase cluster | Threonine synthase cluster |
| Threonylcarbamoyladenosine | Threonylcarbamoyladenosine |
| Translation elongation factors, bacterial | Translation elongation factors, bacterial |
| Translation initiation factors, bacterial | Translation initiation factors, bacterial |
| Translation termination factors, bacterial | Translation termination factors, bacterial |
| Trans-translation by stalled ribosomes | Trans-translation by stalled ribosomes |
| Tricarboxylate transport system | Tricarboxylate transport system |
| Trk and Ktr potassium uptake systems | Trk and Ktr potassium uptake systems |
| tRNA aminoacylation, Ala | tRNA aminoacylation, Ala |
| tRNA aminoacylation, Arg | tRNA aminoacylation, Arg |
| tRNA aminoacylation, Asn | tRNA aminoacylation, Asn |
| tRNA aminoacylation, Asp | tRNA aminoacylation, Asp |
| tRNA aminoacylation, Cys | tRNA aminoacylation, Cys |
| tRNA aminoacylation, Gln | tRNA aminoacylation, Gln |
| tRNA aminoacylation, Glu | tRNA aminoacylation, Glu |
| tRNA aminoacylation, Gly | tRNA aminoacylation, Gly |
| tRNA aminoacylation, His | tRNA aminoacylation, His |
| tRNA aminoacylation, Ile | tRNA aminoacylation, Ile |
| tRNA aminoacylation, Leu | tRNA aminoacylation, Leu |
| tRNA aminoacylation, Lys | tRNA aminoacylation, Lys |
| tRNA aminoacylation, Met | tRNA aminoacylation, Met |
| tRNA aminoacylation, Phe | tRNA aminoacylation, Phe |
| tRNA aminoacylation, Pro | tRNA aminoacylation, Pro |
| tRNA aminoacylation, Ser | tRNA aminoacylation, Ser |
| tRNA aminoacylation, Thr | tRNA aminoacylation, Thr |
| tRNA aminoacylation, Trp | tRNA aminoacylation, Trp |
| tRNA aminoacylation, Tyr | tRNA aminoacylation, Tyr |
| tRNA aminoacylation, Val | tRNA aminoacylation, Val |
| tRNA nucleotidyltransferase | tRNA nucleotidyltransferase |
| tRNA thiolation | tRNA thiolation |
| Tryptophan synthesis | Tryptophan synthesis |
| Twin-arginine translocation system | Twin-arginine translocation system |
| Two cell division clusters relating to chromosome partitioning | Two cell division clusters relating to chromosome partitioning |
| Undecaprenyl-diphosphatases | Undecaprenyl-diphosphatases |
| Universal GTPases | Universal GTPases |
| Universal stress protein family | Universal stress protein family |
| Urea cycle | Urea cycle |
| Urease subunits | Urease subunits |
| VraTSR and LiaFSR three-component regulatory systems | VraTSR and LiaFSR three-component regulatory systems |
| Wall polysaccharide pyruvylation | Wall polysaccharide pyruvylation |
| YrdC-YciO-Sua5 and associated protein families | YrdC-YciO-Sua5 and associated protein families |
